# A Global Collaborative Comparison of SARS-CoV-2 Antigenicity Across 15 Laboratories

**DOI:** 10.3390/v16121936

**Published:** 2024-12-18

**Authors:** Polina Brangel, Sina Tureli, Barbara Mühlemann, Nicole Liechti, Daniel Zysset, Olivier Engler, Isabel Hunger-Glaser, Ioana Ghiga, Giada Mattiuzzo, Isabella Eckerle, Meriem Bekliz, Annika Rössler, Melanie M. Schmitt, Ludwig Knabl, Janine Kimpel, Luis Fernando Lopez Tort, Mia Ferreira de Araujo, Any Caroline Alves de Oliveira, Braulia Costa Caetano, Marilda Mendonça Siqueira, Matthias Budt, Jean-Marc Gensch, Thorsten Wolff, Tarteel Hassan, Francis Amirtharaj Selvaraj, Tandile Hermanus, Prudence Kgagudi, Carol Crowther, Simone I. Richardson, Jinal N. Bhiman, Penny L. Moore, Samuel M. S. Cheng, John K. C. Li, Leo L. M. Poon, Malik Peiris, Victor M. Corman, Christian Drosten, Lilin Lai, Taweewun Hunsawong, Kamonthip Rungrojcharoenkit, Jindarat Lohachanakul, Alex Sigal, Khadija Khan, Volker Thiel, G. Tuba Barut, Nadine Ebert, Anna Z. Mykytyn, Irene Owusu Donkor, James Odame Aboagye, Prince Adom Nartey, Maria D. Van Kerkhove, Jane Cunningham, Bart L. Haagmans, Mehul S. Suthar, Derek Smith, Lorenzo Subissi

**Affiliations:** 1 World Health Organization, 1202 Geneva, Switzerland; 2 Centre for Pathogen Evolution, University of Cambridge, Cambridge CB3 0FD, UK; 3 Charité–Universitätsmedizin Berlin, Corporate Member of Freie Universität Berlin, Humboldt-Universität zu Berlin, Institute of Virology, Charitéplatz 1, 10117 Berlin, Germany; 4 German Centre for Infection Research (DZIF), Associated Partner Site Charité, Charitéplatz 1, 10117 Berlin, Germany; 5 WHO BioHub Facility, Spiez Laboratory, 3700 Spiez, Switzerland; 6 Medicines and Healthcare Products Regulatory Agency, London SW1W 9SZ, UK; 7 Department of Medicine, University of Geneva, 1205 Geneva, Switzerland; 8 Division of Infectious Diseases, Geneva University Hospitals, 1205 Geneva, Switzerland; 9 Geneva Centre for Emerging Viral Diseases, University Hospitals of Geneva and University of Geneva, 1205 Geneva, Switzerland; 10 Institute of Virology, Department of Hygiene, Microbiology and Virology, Medical University of Innsbruck, 6020 Innsbruck, Austria; 11 Tyrolpath Obrist Brunhuber GmbH, 6511 Zams, Austria; 12 Laboratory of Respiratory, Exanthematous and Enteric Viruses and Viral Emergencies (LVRE), Oswaldo Cruz Institute (IOC-Fiocruz), Rio de Janeiro 21.040-900, Brazil; 13 Laboratory of Molecular Virology, Department of Biological Sciences, CENUR Litoral Norte, Universidad de la República, Salto 50000, Uruguay; 14 Unit 17 “Influenza and Other Respiratory Viruses”, Robert Koch Institut, 13353 Berlin, Germany; 15 Reference Laboratory for Infectious Diseases, Purelab, Sheikh Khalifa Medical City, Abu Dhabi 51900, United Arab Emirates; 16 SAMRC Antibody Immunity Research Unit, School of Pathology, University of the Witwatersrand, Johannesburg 2001, South Africa; 17 National Institute for Communicable Diseases of the National Health Laboratory Services, Johannesburg 2131, South Africa; 18 Centre for the AIDS Programme of Research in South Africa, Durban 4001, South Africa; 19 School of Public Health, LKS Faculty of Medicine, The University of Hong Kong, Hong Kong SAR, China; 20 Department of Pediatrics, Center for Childhood Infections and Vaccines, Emory Vaccine Center, Emory University School of Medicine, Atlanta, GA 30322, USA; 21 Walter Reed Army Institute of Research-Armed Forces Research Institute of Medical Sciences, Bangkok 10500, Thailand; 22 The Lautenberg Center for Immunology and Cancer Research, Faculty of Medicine, Hebrew University of Jerusalem, Jerusalem 91904, Israel; 23 Africa Health Research Institute, Durban 4013, South Africa; 24 Multidisciplinary Center for Infectious Diseases, University of Bern, 3012 Bern, Switzerland; 25 Institute of Virology and Immunology, Mittelhäusern and Bern, 3012 Bern, Switzerland; 26 Erasmus Medical Centre, 3015 Rotterdam, The Netherlands; 27 Medical and Scientific Research Centre, University of Ghana Medical Centre, Accra P.O. Box LG 25, Ghana; 28 Noguchi Memorial Institute for Medical Research, University of Ghana, Legon P.O. Box LG 581, Ghana

**Keywords:** neutralisation, antigenicity, SARS-CoV-2, COVID-19, Bayesian model, global surveillance

## Abstract

Setting up a global SARS-CoV-2 surveillance system requires an understanding of how virus isolation and propagation practices, use of animal or human sera, and different neutralisation assay platforms influence assessment of SARS-CoV-2 antigenicity. In this study, with the contribution of 15 independent laboratories across all WHO regions, we carried out a controlled analysis of neutralisation assay platforms using the first WHO International Standard for antibodies to SARS-CoV-2 variants of concern (source: NIBSC). Live virus isolates (source: WHO BioHub or individual labs) or spike plasmids (individual labs) for pseudovirus production were used to perform neutralisation assays using the same serum panels. When comparing fold drops, excellent data consistency was observed across the labs using common reagents, including between pseudovirus and live virus neutralisation assays (RMSD of data from mean fold drop was 0.59). Utilising a Bayesian model, geometric mean titres and assay titre magnitudes (offsets) can describe the data efficiently. Titre magnitudes were seen to vary largely even for labs within the same assay group. We have observed that overall, live Microneutralisation assays tend to have the lowest titres, whereas Pseudovirus Neutralisation have the highest (with a mean difference of 3.2 log2 units between the two). These findings are relevant for laboratory networks, such as the WHO Coronavirus Laboratory Network (CoViNet), that seek to support a global surveillance system for evolution and antigenic characterisation of variants to support monitoring of population immunity and vaccine composition policy.

## 1. Introduction

The evolution of SARS-CoV-2, particularly the emergence of the Omicron variant, has had profound implications for global health and pandemic management. Understanding the mechanisms driving viral evolution, including genetic variation, selective pressures, and antibody escape, is crucial for developing effective interventions [1].

SARS-CoV-2 surveillance and research has revealed unprecedented numbers of genomic sequences, shedding light on evolutionary events that had previously been inferred indirectly or had gone undetected. These events include the emergence of variants with distinct phenotypes such as transmissibility, severity, and immune escape [1].

The antibody escape capacity of Omicron subvariants poses challenges for neutralising antibody efficacy generated by vaccines with the initial composition or convalescent exposure to a prior variant of concern (VOC) [2]. The XBB.1.5 variant, which possesses 40 amino acid substitutions in the spike protein compared to the index virus, features enhanced viral fitness, transmission, and capacity to escape neutralising antibodies. Notably, mutations such as E484A, K417N, and N501Y are associated with a higher risk of antibody escape [3]. Understanding the structural and functional changes in emerging variants and their sub-lineages is essential for developing targeted therapeutics and vaccines [3].

Variations within VOCs can significantly impact viral transmissibility, immune escape potential, and vaccine effectiveness. Even minor alterations in key regions of the spike protein may influence antibody neutralisation and the effectiveness of immunological countermeasures. Hence, assessing the antibody escape capabilities of emerging variants of SARS-CoV-2 is paramount for guiding vaccination strategies, informing public health measures, optimising treatment options, and enhancing global surveillance efforts to control the ongoing COVID-19 pandemic. Understanding how variants evade the antibody response generated by vaccines is essential for evaluating vaccine efficacy and determining the need for vaccine updates. In addition, monitoring cross-neutralisation of emerging variants contributes to global surveillance efforts aimed at detecting and responding to potential threats of emerging SARS-CoV-2 variants that may escape antibodies from infection and/or vaccination. This allows for the implementation of targeted interventions to prevent their spread and minimise their impact on populations worldwide [4].

As neutralising antibody titres have been shown to be predictive of immune protection from symptomatic disease, neutralisation assays are crucial for the rapid and reliable assessment of antibody escape of emerging variants [5]. These assays provide a rapid and sensitive means of evaluating the potency of an antibody response generated by vaccination or natural infection. This rapid assessment is essential for informing vaccine development strategies, optimising vaccine formulations, and guiding public health responses to emerging variants [6]. Since the onset of the pandemic, a number of different SARS-CoV-2 neutralisation assays based on either authentic replication competent SARS-CoV-2 isolates, lentiviral-based pseudoviruses, or vesicular stomatitis virus (VSV)-based chimeric viruses have been used and established. However, limited studies have directly compared the results of different assays using the same serum samples [7,8,9,10]. Establishing the comparability of neutralisation data derived from various laboratories utilising different assays and human sera lays the groundwork for a robust global surveillance system integrating data from diverse sources [11,12]. Additionally, ongoing surveillance of SARS-CoV-2 variants, including their prevalence in different regions, informs vaccine development strategies [13].

To gain deeper insights into the variability of neutralisation assays conducted across different laboratories employing diverse protocols, we conducted a comprehensive analysis utilising data from 15 laboratories spanning 12 countries. Our study aimed to establish a collaborative laboratory framework to assess the antigenic variation of SARS-CoV-2 variants, specifically focusing on the XBB.1.5 variant as a proof of concept. This included evaluating the comparability of neutralisation assays across different laboratories using the same plasma and analysing the variability in immune responses based on unique epidemiological contexts to gain insights into the virus’s immunological landscape. Some labs have also performed titrations using Lentivirus-based pseudoviruses, their own in-house live viruses, and in-house human sera. Through this multifaceted approach, we aimed to elucidate assay comparability on a global scale and provide insights into the dynamics of SARS-CoV-2 immune responses.

## 2. Methods

### 2.1. Study Design

This study was designed to harmonise the antigenic characterisation through neutralisation assays, reflecting different methodologies commonly employed in the field, before their application in evaluating the immune escape potential of emerging SARS-CoV-2 variants. Assays evaluated included the Plaque Reduction Neutralisation Test (PRNT), Focus Reduction Neutralisation Test (FRNT), Microneutralisation (MicroNeut), and Lentivirus-based Pseudovirus Neutralisation Test (PNT). Immune responses were quantified using standardised units specific to each assay method. Furthermore, 50% neutralisation dilution (ND50) was utilised for MicroNeut, and for PRNT, 50% neutralisation titre (NT50). Fold drop was calculated by sampling the difference between wild-type log2 titres and other antigens’ log2 titres within the Bayesian model framework (see Mathematical Methods Section in SI).

The selection of these assays was guided by recommendations from the WHO Technical Advisory Group for Virus Evolution (TAG-VE) and outlined in Figure S23. Adding to the existing SARS-CoV-2 reference laboratory network, which has now transitioned to Coronavirus Network (CoViNet), over 35 laboratories were identified by a small internal steering group on the basis of their experience working in SARS-CoV-2 serology and were invited to participate in this study. A total of 22 reference laboratories expressed interest in participation and completed a competency survey to evaluate their proficiency and capacity to effectively perform the required assays. Criteria for inclusion encompassed laboratory capacity, competency to execute the specified assays, and geographical distribution across all six WHO regions. Seventeen laboratories were initially enrolled, out of which sixteen laboratories successfully completed the legal enrolment and conducted the experimental work and are included in this report. Two laboratories were excluded due to incomplete legal enrolment procedures. The final cohort of 16 participating laboratories exhibited a diverse representation, spanning across 12 countries across all six WHO regions (6 in the European region, 3 in the African region, 3 in the American region, 1 in the Eastern Mediterranean region, 1 in the Southeast Asia region, and 2 in the Western Pacific region; Figure S23B).

Out of 16 participating laboratories in this work, 15 laboratories successfully conducted the experimental work for the comparability analysis. Results from one laboratory were excluded from this exercise due to insufficient amounts of viral isolates to repeat the experimental work. Two other labs had to repeat and extend their titrations, in one case due to an upper limit of detection being too low for most antigens and in the other case producing unexpected results possibly also due to low titres of propagated virus isolates. One lab which also could not propagate enough virus stocks only performed a Pseudovirus Neutralisation assay after generating their own pseudoviruses according to the protocol specified below. The laboratories engaged in the assessment, with 7, 5, 6, and 2 laboratories utilising PRNT, FRNT, MicroNeut, and PNT, respectively. Notably, 4 out of the 16 laboratories performed parallel analyses using two different methods. Each participating laboratory received a comprehensive set of materials, including a study protocol, Annex, six distinct viral isolates (as detailed in Table S1), a reference plasma pool with neutralisation activity against Omicron variants, as well as a negative reference plasma produced and characterised by the NIBSC.

### 2.2. Viral Isolates and Standardised Control Reference Panel

The concept of the work in this session was to test the comparability of neutralising results across laboratories using the same shared reagents, including viral isolates and reference sera panels. The selection of variants for this assessment was determined based on the epidemiological exposure in the global population as well as the genomic difference. A total of 6 viral isolates were selected, produced by WHO BioHub Facility, and distributed among 16 laboratories. The following variants were selected for the analysis: Alpha, Beta, Delta, BA.1, BA.5.2, and XBB.1.5 using GISAID accession numbers EPI_ISL_3147384, EPI_ISL_2401142, EPI_ISL_5394579, EPI_ISL_7456457, EPI_ISL_12268493.2, and EPI_ISL_16760602, respectively. These isolates were produced in VeroE6/TMPRSS2 and/or Calu-3 (VERO6T/C-3) cell lines. Some labs have only titrated against a subset of the antigens. These labs also received two types of plasma pool, NIBSC 21/338 [14] and NIBSC 20/142 [15]. At the time of the assessment, Q1 2023, XBB.1.5 was an emerging variant with a rapid rise globally.

Well-characterised, traceable standards and negative reference materials were provided for validation and harmonisation of test results across laboratories. The first WHO International Standard for antibodies to SARS-CoV-2 variants of concern (21/338) was created from a convalescent plasma pool from 261 donors. All donors were exposed to SARS-CoV-2 via infection (pre-Omicron variants) and vaccination (Index variant). Previous to this study, the 21/338 neutralisation activity was characterised against Alpha, Beta, Delta, and Omicron BA.1 and BA.2 variants of concern [16]. A total of 1 vial with 0.25 mL freeze-dried material was provided. A negative plasma pool (NIBSC code 20/142) collected from healthy UK individuals prior 2019 was also provided.

### 2.3. Experimental Methods

#### 2.3.1. Preparation of SARS-CoV-2 Isolates for the WHO BioHub Facility

All work was performed in accordance with Swiss law and the biosafety guidelines of the Spiez Laboratory in the containment level 3 facility. All samples were handled in biosafety cabinets class II (BSCII). Different SARS-CoV-2 were separated at all times and handled in different BSCs. All work performed is permitted by the Swiss Federal Office of Public Health under the permission numbers A2100984 and A151534. The accession numbers of the viral isolates used in this study can be found in Table S2.

#### 2.3.2. Clinical Specimens

Clinical specimens (2021-WHO-LS-001, 2021-WHO-LS-003, 2021-WHO-LS-016) were imported to the level 3 containment laboratory and thawed. For quality assurance, 100 uL of the samples was directly inactivated in 900 µL Qiazol (Qiagen, Hilden, Germany) according to the manufacturer’s protocol for RNA extraction and whole-genome sequencing. In parallel, 100 ul of the samples was inoculated on either 1× T75 cell culture flask (TPP) containing 3 × 10^6^ VeroE6 TMPRSS2 (NIBSC, 100978) cells and 5 mL of growth medium with 2% FBS and 1 mg/mL Geneticin or 1× T75 cell culture flask containing 2.4 × 10^6^ Calu-3 (ATCC, HTB-55) cells and 5 mL of growth medium with 2% FBS. The culture flasks were transferred in a GENbox (Biomerieux) supported with CO_2_ (Biomerieux) and water for humidification and incubated for 1 h at 37 °C. After the incubation, the culture flasks were removed from the GENbox and the growth medium was removed. The cells then were washed 1× with sterile PBS, and 25 mL of fresh growth medium was added to the flasks. The cell culture flasks were then again transferred to the GENbox, supported with CO_2_ and H_2_O, and incubated at 37 °C for 2–5 days. Virus growth was monitored daily by microscopy for cytopathic effect (CPE) and PCR. When CPE > 70%, the medium containing SARS-CoV-2 variants was harvested by transferring into 50 mL conical tubes and centrifuged for 10 min at 500 g. The supernatant was then transferred to new 50 mL conical tubes, and 100 µL of the supernatant was inactivated using Qiazol as described above. Remaining supernatant was distributed to 1.8 mL cryotubes (Sarstedt) at 500 µL/tube and transferred to −80 °C for storage.

#### 2.3.3. Virus Isolates

Propagation procedures for viral isolates (2022-WHO-LS-014, 2022-WHO-LS-028, 2023-WHO-LS-001) are in accordance with the procedures for clinical specimens as described above with the following adaptations. After the first 1 h incubation, the inoculation medium was not removed and cells were not washed with PBS. Simply, 20 mL of fresh growth medium was added to the cell culture flasks following the incubation for 2–5 days.

#### 2.3.4. Titre Determination by TCID50

For titre determination, 96-well plates (TPP) were seeded with 2 × 10^5^ VeroE6 TMPRSS2 cells/well on day prior the assay. The following day, in the containment laboratory level 3, one aliquot/sample was thawed and a 10-fold dilution up to 10^−9^ was prepared. Six replicates of each dilution were then transferred to the prepared 96-well plates and incubated at 37 °C (in GENbox boxes supported with CO_2_ and H_2_O) for 3 days. After the incubation, the supernatants of the plates were removed and 70 µL/well of crystal violet, already containing formaldehyde and ethanol, was added. The plates were then incubated for 10 min at RT before the crystal violet was removed. The plates were then sealed with foil and pictures were taken. TCID50 was then calculated according to Spearman and Karber [17,18].

#### 2.3.5. Next-Generation Sequencing

Inactivated RNA of original material and cultivated virus was extracted using the RNeasy Universal Plus kit (Qiagen) according to the manufacturer’s instructions. Extracted RNA was subjected to whole-genome sequencing using an Ion AmpliSeq™ SARS-CoV-2 Insight Research Panel (ThermoFisher, Reinach, Switzerland) according to the manufacture’s protocol. Briefly, RNA was reverse-transcribed using an Ion Torrent™ NGS Reverse Transcription Kit (ThermoFisher) followed by automated library preparation on an Ion Chef system using 16 target amplification cycles. Final libraries were loaded automatically on an Ion 530™ Chip using the Ion Chef system and the chip was sequenced on an Ion S5 Plus sequencer according to the manufacturer’s instructions. Data analysis was performed using Torrent Suite™ software version 5.18.1 with the following plugins: SARS_CoV_2_coverageAnalysis v5.16.0.4, SARS_CoV_2_variantCaller v5.16.0.4, SARS_CoV_2_annotateSnpEff v5.16.0.4, generate Consensus v5.16.0.4. Quality of consensus sequences was checked manually, and sequences of cultured virus were checked for introduction of additional mutations during culturing process.

#### 2.3.6. Sample Storage and Shipping

All samples were stored at −80 °C until shipment. For the shipment, the samples were sluiced out of the containment facility and packed according to IATA packing instructions p620. For transfer, all samples were packed with dry ice, and temperature was monitored. If required, dry ice was replenished during transfer.

### 2.4. Individual Laboratory Assays

#### 2.4.1. Africa Health Research Institute

##### Virus Expansion

All work with live virus was performed in biosafety level 3 containment using protocols for SARS-CoV-2 approved by the Africa Health Research Institute Biosafety Committee. VeroE6-TMPRSS2 cells were seeded at 4.5 × 10^5^ cells in a 6-well plate and incubated for 18–20 h pre-infection. The virus stock was diluted 1:5 with Dulbecco’s Modified Eagle Medium (DMEM, Gibco 41965-039) containing 10 mM of hydroxyethylpiperazine ethanesulfonic acid (HEPES, Lonza, 17-737E), 1 mM sodium pyruvate (Gibco, 11360-039), 2 mM L-glutamine (Lonza BE17-605E), and 0.1 mM non-essential amino acids (Lonza 13-114E) growth medium containing no foetal bovine serum and filtered through a 0.45 μm filter. After one Dulbecco’s phosphate-buffered saline (DPBS) wash, the sub-confluent cell monolayer was inoculated with 500 μL diluted sample and incubated for 2 h. Wells were then filled with 3 mL complete growth medium containing 10% foetal bovine serum. After 2 days of infection (completion of passage 1 (P1)), supernatant was collected and cells were trypsinised, centrifuged at 300× *g* for 3 min, and resuspended in 5 mL growth medium. All infected cells and supernatant were added to VeroE6-TMPRSS2 cells that had been seeded at 1.5 × 10^5^ cells per mL, 20 mL total, 18–20 h earlier in a T75 flask for cell-to-cell infection. The coculture was incubated for 1 h, and the flask was filled with 20 mL of complete growth medium and incubated for 2 days. The viral supernatant from this culture (passage 2 (P2) stock) was used for experiments.

##### Focus Reduction Neutralisation Test

Test was performed as described in reference [19,20]. VeroE6-TMPRSS2 cells were plated in a 96-well plate (Corning) at 30,000 cells per well 1 day pre-infection. For the XBB panel, plasma was separated from EDTA-anticoagulated blood by centrifugation at 500× *g* for 10 min and stored at −80 °C. Aliquots of plasma samples were heat-inactivated at 56 °C for 30 min and clarified by centrifugation at 10,000× *g* for 5 min. Virus stocks were used at approximately 100 focus-forming units per microwell and added to diluted plasma in neutralisation assays. Antibody–virus mixtures were incubated for 1 h at 37 °C, 5% CO_2_. Cells were infected with 100 μL of the virus–antibody mixtures for 1 h, then 100 μL of a 1× RPMI 1640 (Sigma-Aldrich, St. Louis, MO, USA, R6504), 1.5% carboxymethylcellulose (Sigma-Aldrich, C4888) overlay was added without removing the inoculum. Cells were fixed 20 h post-infection using 4% PFA (Sigma-Aldrich, P6148) for 20 min. Foci were stained with a rabbit anti-spike monoclonal antibody (BS-R2B12, GenScript A02058) at 0.5 μg/mL in a permeabilisation buffer containing 0.1% saponin (Sigma-Aldrich, S7900), 0.1% bovine serum albumin (Biowest, P6154), and 0.05% Tween-20 (Sigma-Aldrich, P9416) in PBS for 2 h at room temperature with shaking, then washed with wash buffer containing 0.05% Tween-20 in PBS. Secondary goat anti-rabbit HRP conjugated antibody (Abcam ab205718) was added at 1 μg/mL and incubated for 2 h at room temperature with shaking. TrueBlue peroxidase substrate (SeraCare 5510-0030) was then added at 50 μL per well and incubated for 20 min at room temperature. Plates were imaged in an ImmunoSpot Ultra-V S6-02-6140 Analyzer ELISPOT instrument (Immunospot, Cleveland, OH, USA) with BioSpot Professional built-in image analysis (C.T.L).

##### Statistics and Fitting

All statistics and fitting were performed using custom code in MATLAB v.2019b. Neutralisation data were fit to Tx = 1/1 + (D/ID_50_). Here, Tx is the number of foci at plasma dilution D normalised to the number of foci in the absence of plasma on the same plate. ID_50_ is the plasma dilution giving 50% neutralisation. FRNT_50_ = 1/ID_50_. Values of FRNT_50_ < 1 are set to 1 (undiluted), the lowest measurable value. We note that the most concentrated plasma dilution was 1:25 and the most dilute plasma sample was 1:3200. Therefore, FRNT_50_ < 25 and FRNT50 > 3200 were extrapolated from the fit. Script in MATLAB v.2019b to fit neutralisation data for FRNT50 is available on GitHub (https://github.com/sigallab/NatureMarch2021, accessed on 11 December 2024).

#### 2.4.2. Center for Disease Control—USA

##### Plaque Reduction Neutralisation Titre Assay (PRNT)

Each SARS-CoV-2 P2 virus analysed was titrated and diluted to 4000–8000 focus-forming units (FFUs) per ml in DMEM supplemented with 2% heat-inactivated foetal [21] bovine serum (HyClone, Logan, UT, USA) and 1× Anti-Anti (Gibco). Human serum samples were heat-inactivated at 56 °C for 30 min and serially diluted 3-fold or 4-fold for 7 dilutions in sextuplicate in 96-well untreated round-bottom plates. Serum dilution was started at 1:5 for post-primary series sera, 1:10 for post-third-dose sera, or 1:20 for post-bivalent sera. Diluted sera were mixed with equal volume of diluted virus and incubated at room temperature for one hour. After removing the cell culture medium from 96-well tissue culture plates containing confluent Vero/TMPRSS2 cells, cells were inoculated with 50 µL virus–sera mixture in each well. The plates were incubated at 37 °C with 5% CO_2_ for 1 h, overlaid with 100 µL 0.75% methylcellulose (Sigma-Aldrich) in DMEM supplemented with 10% heat-inactivated foetal bovine serum (HyClone) and 1× Penicillin–Streptomycin (Gibco, Waltham, MA, USA), and then incubated at 33 °C with 5% CO_2_ for 20–22 h. The next day, methylcellulose was removed from each well, and 100 µL 70% ethanol was added for 10 min to fix and permeabilise the cells. Cells were washed with PBS, blocked with SuperBlock Blocking Buffer (Thermo Scientific, Waltham, MA, USA) for 30 min at room temperature, and stained with SARS/SARS-CoV-2 Coronavirus Nucleocapsid Monoclonal Antibody (Thermo Scientific, MA5-29981) at 1:4000 dilution at 4 °C overnight. After washing with PBS, cells were stained with IgG (H + L) highly cross-adsorbed secondary antibody with Alexa Fluor™ Plus 647 (Thermo Scientific, A32728) at 1:400 dilution for 1 h at room temperature. All plates were imaged using a CellInsight CX5 High-Content Screening Platform (Thermo Scientific) under 2× magnification with the same setting. Fluorescent virus foci were identified and quantified using Cellomics Scan Version 6.6.2 (Thermo Scientific, Build 8533) and exported to Excel and R for downstream analysis. FRNT50 values were calculated using a three-parameter log-logistic function (LL.3) in R. In cases where the hill constant was fit outside of the range from 0.5 to 2, a two-parameter fit while fixing the hill constant to 1 was used to estimate the FRNT50 values. The R script has been deposited in [22] (accessed on 15 January 2023).

#### 2.4.3. Charité–Universitätsmedizin Berlin

##### PRNT Assay

Vero E6 (ATCC CRL-1586) and Calu-3 (HTB-55) cells were maintained at 37 °C, 5% CO_2_ in culture medium Dulbecco’s Modified Eagle’s Medium (DMEM, ThermoFisher Scientific) supplemented with 10% foetal bovine serum (FBS, ThermoFisher Scientific), 1% non-essential amino acids (ThermoFisher Scientific), and 1% sodium pyruvate 100 mM (NaP, ThermoFisher Scientific). All cell lines tested negative for Simian virus 5 (*Orthorubulavirus mammalis*) and mycoplasma contaminations. Plaque reduction neutralisation tests were performed as described in [23,24]. Briefly, Vero E6 cells (1.6 × 10^5^ cells/well) were seeded in 24-well plates and incubated for ~24 h. Sera were serially diluted in OptiPro medium and mixed with medium containing 100 PFU of the respective virus, incubated at 37 °C for one hour, and then added to the Vero E6 cells in duplicate. After a further hour at 37 °C, supernatants were discarded and the cells washed once with PBS and supplemented with 1.2% Avicel solution in DMEM. After three days at 37 °C, the supernatants were removed and the plates were fixed using a 6% formaldehyde/PBS solution and stained with crystal violet. All serum dilutions were tested in duplicates. Plaques were counted for each well.

#### 2.4.4. Emory University

##### Viruses and Cells

VeroE6-TMPRSS2 cells were generated and cultured as previously described [25]. All variants were plaque-purified and propagated once in VeroE6-TMPRSS2 cells to generate working stocks. Viruses were deep-sequenced and confirmed as previously described [26].

##### Focus Reduction Neutralisation Test

VeroE6-TMPRSS2 cells were generated and cultured as previously described [25]. Serum samples in duplicate were 3-fold diluted in 8 serial dilutions using DMEM with an initial dilution of 1:10. Serially diluted samples were mixed with an equal volume of SARS-CoV-2 (100–200 foci per well). The virus–serum mixtures were incubated at 37 °C for 1 h in a round-bottom 96-well culture plate. After 1 h incubation, the virus–serum mixture was added to VeroE6-TMPRSS2 cells and incubated at 37 °C for an additional hour. Post-incubation, the mixture was removed from cells, and 100 μL of prewarmed 0.85% methylcellulose overlay was added to each well. Plates were incubated at 37 °C for 18 to 40 h (depending on variants). After the appropriate incubation time, the methylcellulose overlay was removed, and cells were washed with PBS and fixed with 2% paraformaldehyde for 30 min. Following fixation, cells were washed twice with PBS and permeabilised using a permeabilisation buffer for at least 20 min. Cells were incubated with an anti-SARS-CoV-2 spike primary antibody directly conjugated to Alexa Fluor-647 (CR3022-AF647) overnight at 4 °C. Cells were then washed twice with 1X PBS and imaged on an ELISPOT reader (CTL Analyzer). Antibody neutralisation was quantified by counting the number of foci for each sample using the Viridot program. The neutralisation titres were calculated as follows: 1—(ratio of the mean number of foci in the presence of sera and foci at the highest dilution of the respective serum sample). Each sample was tested in duplicate. The FRNT-50 titres were interpolated using a 4-parameter non-linear regression in GraphPad Prism 10.1.2.

#### 2.4.5. Erasmus Medical Center

##### FRNT

The FRNT50 assay was performed as described previously [27]. Calu-3 cells, maintained in Opti-MEM I (1×) + GlutaMAX (Gibco) supplemented with 10% FBS, penicillin (100 IU/mL), and streptomycin (100 IU/mL), were seeded in 96-well plates to reach full confluency prior to experiments. Sera were heat-inactivated at 56 °C for 30 min and serially diluted in 60 μL of Opti-MEM I (1×) + GlutaMAX. An amount of 400 foci forming units was added per well as 60 μL in Opti-MEM I (1×) + GlutaMAX and plates were incubated at 37 °C for one hour. An amount of 100 μL of the virus and serum mix was added to Calu-3 cells and incubated for 8 h at 37 °C in a humidified CO_2_ incubator. Plates were fixed in formalin and cells were permeabilised with ethanol. Plates were washed in PBS and stained with rabbit anti-nucleocapsid (Sino Biological, Eschborn, Germany; 1:2000) in PBS containing 0.1% bovine serum albumin (BSA). Cells were washed in PBS and stained with goat anti-rabbit Alexa Fluor 488 (Invitrogen, Waltham, MA, USA; 1:4000) in PBS containing 0.1% BSA. Cells were next stained with Hoechst (Thermo Fisher Scientific) and washed with PBS. Imaging was performed on an Opera Phenix spinning disk confocal HCS system (PerkinElmer, Groningen, The Netherlands), and non-linear regression in GraphPad Prism 9 software was used to determine the serum dilution inhibiting 50% of infection. All SARS-CoV-2 work described above was performed in a Class II Biosafety Cabinet under BSL-3 conditions at Erasmus Medical Center.

#### 2.4.6. Fundação Oswaldo Cruz-Rio de Janeiro

##### PRNT

PRNT was used to determine the infectious titre after neutralisation as previously described by [28] with some modifications. Briefly, sera were heat-inactivated at 56 °C for 30 min to inactivate the complement system. Heat-inactivated serum samples were subjected to PRNT_50_/PRNT_90_ in Vero cells (ATCC, CCL 81) maintained in cell culture medium supplemented with foetal bovine serum, sodium bicarbonate, and antibiotics/antimycotics and incubated in 5% CO_2_ atmosphere at 37 °C. Heat-inactivated serum samples were screened in 2–3-day-old Vero CCL-81 cells seeded in 12-well cell culture plates. Sera were mixed with 40–60 PFU of the respective SARS-CoV-2 isolate and incubated at 37 °C for 1 h. Afterward, cell culture medium was removed from the 12-well cell culture plates, each well was inoculated with the virus–serum mixture and incubated at 37 °C for 1 h, and then prewarmed cell culture medium containing 0.5% ultrapure agarose (Sigma-Aldrich) was overlaid. Serum samples were tested in duplicates in serial 2-fold dilutions that ranged from 1:10 to up to 1:320 for their ability to neutralise 40–60 plaque-forming units (PFUs) by each one of the VOCs SARS-CoV-2 reference isolates produced by WHO BioHub Facility (Alpha, Beta, Omicron BA.1, Omicron BA.5, and Omicron XBB.1.5). For each neutralisation experiment, an infection control (no serum) and a reference serum provided by the WHO were used to ensure reproducibility between different experiments. After 48 h of incubation, plates were overlaid with prewarmed cell culture medium containing 0.5% ultrapure agarose and neutral red solution (Sigma-Aldrich), and after 72 h, PFUs were visualised and counted through a transilluminator. Serum samples were considered reactive to SARS-CoV-2 when a serum dilution of at least 1:10 reduced no <50% (PRNT_50_) < 90% (PRNT_90_) of the PFU of SARS-CoV-2, as previously reported [29]. Serum samples that presented PRNT_50_ and/or PRNT_90_ titres > 320 and had enough volume available were retested in higher dilutions to reach endpoint titres. Serum samples were considered seropositive to a specific VOC of SARS-CoV-2 when it had PRNT_50_ and/or PRNT_90_ titre of at least 10. Serum samples were considered seronegative to a specific VOC of SARS-CoV-2 when it had PRNT_50_ and/or PRNT_90_ titre < 10 [28].

##### Individual Serum Samples

Immunocompetent and healthy individual samples (n = 20) consisted of serum samples collected after infection, vaccination, or a combination of both (hybrid immunity) from volunteers in the context of the study “RECOVER-SUS Brazil Network” at the Oswaldo Cruz Foundation (FIOCRUZ). Serum samples were collected from boosted healthy individuals, with two doses of ChAdOx1 nCoV-19 (Oxford/AstraZeneca) vaccine, third booster dose with BNT162b2 (Pfizer/BioNTech), and fourth booster dose of COMIRNATY bivalent vaccine (Pfizer/BioNTech).

#### 2.4.7. Hôpitaux Universitaires de Genève

##### FRNT

FRNT was used to determine the infectious titre after neutralisation. Vero-E6-TMPRSS (Vero-E6 overexpressing TMPRSS2 protease, provided by National Institute for Biological Standards and Controls, NIBSC, Cat. Nr. 100978) cells were seeded at a density of 4 × 10^5^ cells/mL in 96-well cell culture plates. All sera were heat-inactivated at 56 °C for 30 min and serially diluted in Opti-Pro serum-free. Sera were mixed with 50 FFU of the respective SARS-CoV-2 isolate (Alpha, Beta, Omicron BA.1, Omicron BA.5, and Omicron XBB.1.5) and incubated at 37 °C for 1 h. All samples were run in duplicate, and for each neutralisation experiment, an infection control (no serum) and a reference serum provided by the WHO were used to ensure reproducibility between different experiments. Vero-E6-TMPRSS cells were washed 1× with PBS and inoculated with the virus–serum mixture for 1 h. Afterward, the inoculum was removed and 100 µL of the prewarmed medium containing 0.85% methylcellulose was overlaid. After incubation for 16–24 h at 37 °C, 5% CO_2_, the overlay medium was removed, and cells were fixed in 6% formaldehyde solution for at least 1 h at room temperature. Cells were permeabilised with 0.1% Triton X-100 and blocked with 1% BSA (Sigma-Aldrich). Plates were then incubated with a primary monoclonal antibody targeting SARS-CoV-2 nucleocapsid protein (purchased from Geneva Antibody Facility, diluted to 0.3 µg mL^−1^) for 1 h at room temperature and then with peroxidase-conjugated secondary antibody (Jackson ImmunoResearch, 109-036-09, diluted to 1:2000) for 30 min at room temperature. Foci were visualised using True Blue HRP substrate (Avantor) and imaged on an ELISPOT reader (CTL), Mabtech IRIS. We defined a cluster of adjacent cells expressing viral antigen as a focus. Foci were counted in wells inoculated with virus–serum/plasma mixtures and compared to foci counts in infection control wells. The 50% reduction endpoint titres (FRNT_50_) were calculated by fitting a 4-parameter logistics curve with variable slope to the foci counts of each serum using GraphPad Prism version 9.1.0.

##### Individual Serum Samples

Immunocompetent and healthy individual samples (n = 36) consisted of serum samples collected after infection, vaccination, or a combination of both (hybrid immunity) in the context of the prospective observational study “SEROCoV-WORK+/Specchio-COVID19” at the Geneva University Hospitals (HUG). Serum samples were collected from boosted healthy individuals, with three doses of BNT162b2 (Pfizer/BioNTech) or mRNA-1273 (Moderna) vaccine (n = 9). Asymptomatic or undetected infections of the vaccinated-only group were excluded in those samples by testing all specimens for antibodies against SARS-CoV-2 nucleocapsid (Roche Elecsys anti-SARS-CoV-2 N assay), and only specimens with a negative result were used. Convalescent sera from unvaccinated individuals with one (n = 4) or two (n = 5) confirmed SARS-CoV-2 infections were collected. Specimens of individuals with hybrid immunity were collected from adults vaccinated 3 times with BNT162b2 or mRNA-1273 vaccine and with 1 (n = 9) or 2 (n = 9) confirmed SARS-CoV-2 infections. For infected individuals, infection history was assessed by a questionnaire and was indicated by the individuals to be either diagnosed by RT-PCR or Ag-RDT, but no respiratory specimens of the acute infection episode were available and therefore sequencing of the infecting variant was not possible. The infection information (RT-PCR- or Ag-RDT-confirmed diagnosis) was self-reported by the participants. Based on the data from our Swiss national genomic surveillance, the viruses circulating at the time when our participants were last infected were all Omicrons. Written informed consent was obtained from all participants.

#### 2.4.8. Institute of Virology and Immunology-Universität Bern

##### Microneutralisation Titre Assay (MNT)

Two-fold serial dilutions of heat-inactivated immune sera/plasma were prepared in quadruplicates in 96-well cell culture plates using DMEM cell culture medium (50 μL/well). To each well, 50 μL of DMEM containing 100 plaque-forming units (PFU) of SARS-CoV-2 was added and incubated for 60 min at 37 °C. Subsequently, 100 μL mix of serum + virus was added on confluent Vero E6/TMPRSS2 cells in 96-well plates. After 3 days, the cells were fixed for 1 h at room temperature with 4% buffered formalin solution containing 1% crystal violet (Merck, Darmstadt, Germany). Finally, the microtitre plates were stained with crystal violet and the end-titre of immune serum-mediated protection from cytopathic effect was visually assessed. Neutralisation dose 50% (ND50) values were calculated according to the Spearman and Kärber method.

#### 2.4.9. Medical University of Innsbruck

##### FRNT

Focus-forming neutralisation assays were performed as previously described [30]. Briefly, plasma samples were heat-inactivated and four-fold serially diluted. Duplicates of 4-fold plasma dilutions (starting at a 1:16 dilution) were pre-incubated with virus for 1 h, and mixtures were subsequently transferred to 96-well plates containing Vero cells stably overexpressing ACE2 and TMPRSS2 [7] seeded the previous day. Two hours after infection, the inoculum was removed and fresh medium added. Cells were incubated for a further eight hours and subsequently fixed for 10 min with 96% EtOH. Plates were stained using a convalescent serum (1:1000 diluted) and a polyclonal goat anti-Human Alexa Fluor Plus 488-conjugated goat anti-human IgG secondary antibody (1:1000 diluted; Ref. A48276, Invitrogen, Thermo Fisher Scientific, Vienna, Austria). Spots were counted using an immunospot reader. Continuous 50% neutralisation titres were calculated in GraphPad Prism 9.0.1 (GraphPad Software, Inc., La Jolla, CA, USA) using a non-linear regression. Neutralisation titres for in-house virus isolates for part of the samples have been previously reported.

##### Pseudovirus Generation

Laboratories tasked with producing PNT assays were provided with expression plasmids encoding identical spike variants (Alpha, Beta, Delta, BA.1, BA.5.2, and XBB.1.5) as in the live isolates used within this study (see Supplementary Table S2 for accession numbers of spike sequences). Each laboratory adhered to these guidelines to ensure uniformity and consistency in the generated pseudoviruses. By mandating the sequencing of the produced pseudoviruses, this quality control measure aimed to guarantee the reliability of the PNT assays across laboratories. The use of the same accession for pseudovirus production, coupled with subsequent sequencing, ensured a standardised and comparable basis for the neutralisation tests performed across the 15 participating laboratories.

#### 2.4.10. National Institute for Communicable Diseases of South Africa

##### PNT

Briefly, 293T/ACE2.MF cells modified to overexpress human ACE2 (provided by M. Farzan, Scripps Research) were cultured in DMEM (Gibco) containing 10% FBS and 3 μg/mL of puromycin at 37 °C. Cell monolayers were disrupted at confluency by treatment with 0.25% trypsin in 1 mM EDTA (Gibco). The SARS-CoV-2, Wuhan-1 spike, cloned into pCDNA3.1 was mutated using a QuikChange Lightning Site-Directed Mutagenesis kit (Agilent Technologies) and NEBuilder HiFi DNA Assembly Master Mix (NEB) to include lineage-defining mutations for VOCs. Pseudoviruses were produced by cotransfection in 293T/17 cells with a lentiviral backbone (HIV-1 pNL4.luc encoding the firefly luciferase gene) and either of the SARS-CoV-2 spike plasmids with PEIMAX (Polysciences). Culture supernatants were from cells by a 0.45 μM filter and stored at −70 °C. Plasma samples were heat-inactivated and clarified by centrifugation. Pseudovirus and serially diluted plasma/sera were incubated for 1 h at 37 °C. Cells were added at 1 × 10^4^ cells per well after 72 h of incubation at 37 °C. Luminescence was measured using a PerkinElmer Life Sciences Model Victor X luminometer. Neutralisation was measured as described by a reduction in luciferase gene expression after single-round infection of 293T/ACE2.MF cells with spike-pseudotyped viruses. Titres were calculated as the reciprocal plasma dilution (ID_50_) causing 50% reduction of relative light units [31].

#### 2.4.11. Noguchi Memorial Institute for Medical Research

##### PRNT

All samples were tested in duplicates and assay performed in a biosafety level 3 (BSL-3) facility. VERO E6 TMPRSS2 cells were seeded overnight at 3 × 10^5^ in 24-well plates. A 1:20 dilution of the sera 21/338 and 20/142 received from NIBSC was prepared and serially diluted 2-fold. Equal volumes of the SARS-CoV-2 isolate containing 50 PFU were incubated with sera for 1 h at 37 °C. The sera–virus mixture was transferred onto the cells and incubated for 1 h at 37 °C. The sera–virus mixture was discarded and the cells washed. The cells were overlaid with 1.25% methylcellulose with cell culture medium, and incubated for 96 h (4 days). The cells were then fixed with 4% paraformaldehyde (PFA) for 1 hr, washed, and stained with 0.2% crystal violet. Plaques were counted and the ND50 estimated as the reciprocal of the dilutions of the sera.

##### MNT

The virus stock was titred in triplicates in fold dilution on VERO E6 TMPRSS2 cells (2 × 10^4^) to determine the TCID50. A 1:20 dilution of the sera 21/338 and 20/142 received from NIBSC was prepared and serially diluted 2-fold. The sera were incubated with an equal volume of the 100 TCID50 of the virus for 1 h at 37 °C. After the incubation, 100 µL of sera–virus mixture was transferred on the VERO E6 TMPRSS2 cells seeded overnight. The plate was incubated for 1 hr at 37 °C. The mixture was then discarded, cells washed, medium replaced, and sample incubated between 48 and 72 h. The cells were observed for CPE, and percentage cell death was recorded. In addition, cell viability was determined using the MTT assay or the crystal violet and absorbance measured at 590 nm. The values were plotted and ND50 determined as the reciprocal of the dilutions of the sera.

#### 2.4.12. Reference Laboratory for Infectious Disease—Abu Dhabi

##### PRNT

Briefly, about 9 × 10^4^ cells/μL Vero cell lines were seeded in T24 plates in 7% FBS Minimal Essential Medium (MEM) [GIBCO, Geneva, Switzerland] and incubated at 37 °C with 5% CO_2_ until reaching 95% confluence. Six reference variants of SARS-CoV-2 received from the WHO (Table S2) were propagated and stored at −80 °C until used. Sera from NIBSC with codes 21/338 and 20/142, along with patient sera collected at different phases of the pandemic, were serially diluted and 50 PFU of SARS-CoV-2 virus was added to plates and incubated for 1 h at 37 °C with 5% CO_2_ with gentle mixing every 15 min. After removing the inoculum, 700 μL of gel overlay medium was added and plates were incubated at 37 °C with 5% CO_2_ and observed for plaques. Plates were fixed with 3.65% formaldehyde in PBS [MediChem, Steinenbronn, Germany] for 2 h at room temperature, followed by washing with PBS [GIBCO] and stained with crystal violet for plaque visualisation and ND50 were calculated by BioTek Gen5 software given that the serum dilution that reduces plaque number by 50% compared to the control. All the PNRT were performed in the BSL3 facility at RLID-AD.

#### 2.4.13. Robert KochInstitut

##### PRNT

The Plaque Reduction Neutralisation Test was performed in the BSL-3 facility at RKI as described [32]. Briefly, 1.6 × 10^5^ VeroE6 cells were plated in 24-well plates the day before the experiment. Sera 21/338 and 20/142 obtained from the NIBSC were 2-fold serially diluted and incubated with 50 PFU of SARS-CoV-2 isolates in a total volume of 200 µL for 1 h at 37 °C. The mixture was then used to infect the cells for 1 h at 37 °C. After aspiration of the inoculum, cells were grown for three days at 37 °C for SARS-CoV-2 variants Alpha or XBB.1.5 or for four days at 33 °C (for BA.1) or 37 °C (for BA.5.2), respectively, covered with avicel plaque medium (DMEM containing 2% foetal bovine serum, 1.25% Avicel RC-581 (FMC Corporation, Philadelphia, PA, USA), 2 mM glutamine, 100 µg/mL penicillin/streptomycin, 100 µM non-essential amino acids, 1 mM sodium pyruvate, 0.01% DEAE-dextran, 0.05% NaHCO_3_), and stained with crystal violet. The PRNT_50_ titre represents the reciprocal value of the highest serum dilution that reduces plaque number by at least 50% compared to untreated infection, taking into account the additional 1:1 dilution by the virus solution.

##### PNT

Pseudoviruses were generated and neutralisation assay was performed as described [33]. Briefly, 2 × 10^4^ HT1080-ACE2 cells were plated in white solid 96-well plates the day before the experiment. Sera obtained from the NIBSC were 2-fold serially diluted and incubated with 6 × 10^5^ relative light units (RLU) of pseudovirus in a total volume of 120 µL for 1 h at 37 °C. An amount of 100 µL of the mixture was directly added to the cells and incubated for 24 h at 37 °C. Of note, the mutation pattern of the Delta variant was based on EPI_ISL_3220037, which lacks mutations at G142, G1167, and G1219. Infection was then quantified with a Nano-Glo^®^ Luciferase Assay Kit (Promega, Germany). Cells were washed twice with PBS containing 1 mM MgCL_2_ and 1 mM CaCl_2_ and then lysed for 30 min in 25 µL of 1x Passive lysis buffer. An amount of 25 µL of substrate mix was added and incubated for 185 s. Luminescence was detected on a TriStar LB 941 luminometer (Berthold Technologies, Germany) with 0.1 s counting time. ND50 values were calculated by plotting [Agonist] vs. response with variable slope (four parameters) using GraphPad Prism 9.1.0 (GraphPad Software, Inc.). The ND50 titre is given as the serum dilution that yields half of the pseudovirus control RLU (without serum), taking into account the additional 1:1 dilution by the virus solution.

#### 2.4.14. School of Public Health—University of Hong Kong

##### PRNT

All samples were tested in duplicate using 24-well tissue culture plates (TPP Techno Plastic Products AG, Trasadingen, Switzerland) in a biosafety level 3 facility using Vero E6 TMRESS2 cells [34] as previously described [35]. All sera were heat-inactivated at 56 °C for 30 min prior to testing. Serial two-fold dilutions from 1:10 to 1:320 of each serum sample were incubated with 30–40 plaque-forming units of virus for 1 h at 37 °C, and the virus–serum mix was added onto pre-formed cell monolayers and incubated for 1 h at 37 °C in a 5% CO_2_ incubator. The virus–antibody inoculum was then removed and the cell monolayer was overlaid with 1% agarose in cell culture medium. After 3 days of incubation, the plates were fixed and stained. Antibody titres were defined as the highest serum dilution that resulted in ≥ 50% reduction in the number of virus plaques (PRNT50). Samples with titre > 1:320 were further titrated to 1:10,240.

##### MNT

The virus stock was titrated in quadruplicate in 96-well microtitre plates on Vero E6 TMPRSS2 cells in serial 0.5 log10 dilutions (from 0.5 log to 8 log) to obtain 50% tissue culture infectious dose (TCID50). Serial two-fold dilutions of heat-inactivated sera were made, starting with a dilution of 1:10. The serum dilutions were mixed with equal volumes of 200 TCID50 of SARS-CoV-2 as indicated. After 1 h of incubation at 37 °C, 35 μL of the virus–serum mixture was added in quadruplicate to Vero E6 TMPRSS2 cell monolayers in 96-well microtitre plates. After 1 h of adsorption, the wells were emptied, and 150 μL of culture medium was added to each well and the plates incubated for 3 days at 37 °C in a 5% CO_2_ incubator. A virus back-titration was performed with culture medium replacing serum to assess input virus dose. The CPE was read at 3 days post-infection. The highest serum dilution that completely protected the cells from CPE in half of the wells was taken as the neutralising antibody titre.

##### Individual Serum Samples

A total of 21 serum samples were included in this assessment, which were randomly selected from previous cohorts of studies focusing on immunogenicity assessment post-vaccination or in response to BA.2 infections [36,37]. The composition of these serum samples was as follows: individuals infected by BA.2 (N = 3), individuals who were vaccinated and then infected by BA.2 (N = 3), individuals vaccinated with two doses of BNT162b2 (N = 3), individuals vaccinated with two doses of CoronaVac (N = 3), individuals vaccinated with three doses of BNT162b2 (N = 3), individuals vaccinated with three doses of CoronaVac (N = 3), and individuals who received a bivalent BNT162b2 (WT + BA.4/5) booster following three doses of BNT162b2 (N = 3).

#### 2.4.15. Walter Reed Army Institute of Research—Armed Forces Research Institute of Medical Sciences (WRAIR-AFRIMS)

##### Cell Line and Viruses

Vero E6, green monkey kidney epithelial cell line was obtained from ATCC. Cells were grown in Eagle’s Minimum Essential Medium (EMEM, Invitrogen, USA) supplement with 5% heat-inactivated foetal bovine serum (HIFBS, Invitrogen, USA), 1% L-glutamine, 1% P&S, 40 µg/mL gentamicin, and 0.25 µg/mL fungizone at 35 ± 2 °C in a 5% CO_2_ incubator. One-day-old cells were used for measuring or neutralising antibody by Microneutralisation assay. SARS-CoV-2 viruses Omicron BA.1.1.529 (hCoV-19/ZAF/R17343/21/2021), BA.5 (hCoV-19/ZAF/CAP002/2022), and XBB.1.5 (hCoV-19/CH/7782/2023) variants were obtained through WHO BioHub. All were propagated in Vero E6 cells to generate sufficient titres (100TCID50) for the Microneutralisation assay. All isolates were quantitated by tissue culture infectious dose 50 (TCID50) using the Reed–Muench method based on eight replicates per titration.

##### MNT Assay

MNT assay was used to determine neutralising (NT) antibodies against SARS-CoV-2 Omicron variants. All procedures were performed in a BSL-3 laboratory following a standard neutralisation assay using cytopathic effect (CPE)-based colorimetric readouts. Each assay included eight cell control (CC) and virus control (VC) wells. Positive and negative control samples were diluted with 2% HIFBS/EMEM medium (Invitrogen, USA) at 1:10 dilutions before heat inactivation at 56 °C for 30 min and underwent serial four-fold dilution with media starting until 1:163,840. An equal volume of diluted sample was separately incubated with 100 TCID50 of SARS-CoV-2 Omicron variants BA.1.1.529, BA.5, and XBB.1.5 in 37 °C, 5% CO_2_ incubator for 1 h. The 100 µL of serum–virus mixture was inoculated into duplicate wells of Vero E6 cells in 96-well plates and incubated at 37 °C, 5% CO_2_ for 5 days before staining with 0.02% neutral red (Sigma, USA) in 1× PBS (Invitrogen). After an additional incubation at RT for 1 h, lysis solution was added before measuring OD at 540 nm. Percentage of virus infectivity in VC and samples was calculated based on OD of CC: Infectivity (%) = (OD of CC − OD of sample) × 100. The percentage of inhibition was calculated using the following formula: Inhibition (%) = 100 − [(100 × Infectivity of sample)/Infectivity of VC]. The 50% neutralisation titre (NT50) was calculated using log probit analysis. NT50 titre at equal to or higher than 10 was considered as the positive cut-off for a neutralising antibody against a particular SARS-CoV-2 variant [38].

#### 2.4.16. Victorian Infectious Diseases Reference Laboratory

##### Virus Propagation and MNT Assay

Virus propagation and MNT assays were performed in a BSL-3 laboratory. Viruses obtained from the WHO BioHub Facility were propagated either in Vero E6-TMPRSS2 cells (Delta lineage B.1.617 (hCoV-19/LXB/LNS4354796/2021), Omicron BA.5 (hCoV-19/ZAF/CAP002/2022), and XBB.1.5 (hCoV-19/CH/7782/2023)) or Calu-3 cells (Omicron BA.1.1.529 (hCoV-19/ZAF/R17343/2021)). All viruses were stored at −80 °C and quantitated by TCID50 by performing serial 10-fold dilutions in quadruplicate on Vero E6-TMPRSS2 cells, cytopathic effect was read on day 4, and virus titre was calculated using the Reed–Muench method. For the Microneutralisation assay, serial two-fold dilutions of serum were performed in a 96-well microplate, incubated with 100 TCID50 of virus for 1 h at room temperature, and transferred to Vero E6-TMPRSS2 cells in quadruplicate. Plates were incubated at 37 °C, cytopathic effect was read on day 5, and 50% neutralisation titres were calculated using the Reed–Muench method. Geometric mean titres were calculated from two replicate experiments on each sample.

##### Individual Serum Samples

Serum samples (n = 38) fell into four groups: (1) individuals who had received two doses of ancestral SARS-CoV-2 monovalent vaccine (n = 8), (2) individuals who had received three doses of ancestral SARS-CoV-2 monovalent vaccine (n = 8), (3) individuals who had received three doses of ancestral SARS-CoV-2 monovalent vaccine and one dose of an Omicron BA.1 bivalent vaccine (n = 12), and (4) individuals who had received three doses of ancestral SARS-CoV-2 monovalent vaccine followed by an Omicron breakthrough infection (n = 10). All sera were heat-inactivated at 56 °C for 30 min.

##### Production of Reference Sera

A pooled serum sample was prepared from 300 vaccinated individuals in the United Kingdom. These individuals had received vaccinations as part of the national immunisation program. The pooled sera were collected, processed, and subsequently tested for neutralising reactivity against the Omicron variant of SARS-CoV-2.

### 2.5. Mathematical Methods

The geometric mean titre (GMT) estimate, fold drops, and assay-type titre magnitudes were computed using a Bayesian model. The models were written with the PyMC library [39]. The codes for the models and the data used can be found in the repository [40].

#### 2.5.1. Modelling Assay-Wise Effects and GMTs

**Figure 1:**
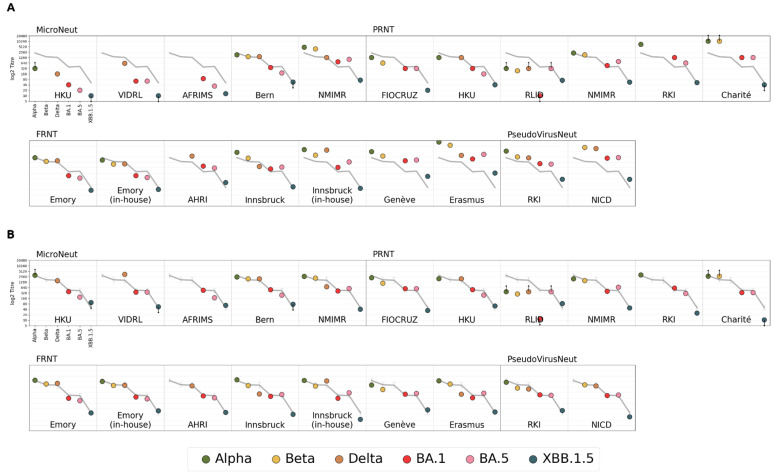
Titres of WHO International Standard NIBSC 21/338 measured in 15 labs using WHO BioHub virus isolates and estimated GMTs. (**A**) Dots in each panel indicate the measured titres of variants (with the colours indicating the variant as shown in the key) by each lab against the International Standard (for multiple repeats, the geometric mean is shown). The grey line indicates the geometric mean titre (GMT) of each of these measurements across all datasets. Up or down arrows, respectively, indicate titres at upper and lower limits of detection. The labels at the bottom of each figure indicate the lab making the measurement (the abbreviations are available in Table S1). Data are grouped according to assay types, which are indicated at the top-left corner for each group (MicroNeut, FRNT, PRNT, or PseudoVirusNeut). Labs labelled as (in-house) or labs using assay type of PseudoVirusNeut have isolated their own viruses for these measurements, whereas all the other measurements were made with virus stocks received from the WHO BioHub (and propagated according to each lab’s specified method, accession numbers 2021-WHO-LS-001, 2021-WHO-LS-003, 2022-WHO-LS-014, 2021-WHO-LS-016, 2022-WHO-LS-028, 2023-WHO-LS-001). (**B**) Each panel in this figure shows the data in Figure 3 after controlling for titre magnitude fitted by the model. The grey line shows the GMT fitted by the model. The bars indicate the 95% high-density interval (HDI) of the posterior for the GMT. The details of the model are given in the SI section Modelling Assay-wise Effects and GM Titres. The number of repeats performed by each lab is given in Table S1.

This model was used in production of the analysis seen in Figure 1 of the Results Section. It aims to represent the log titres measured by each lab (using the common NIBSC 21/338 serum and common antigens) as a sum of some base titres + titre magnitude + noise. Titre magnitude here (defined more explicitly in the model below) can be thought of as the mean (across antigens) titre of a titre curve (in log2 units).

Models similar to this were used in [23], with some of the models there involving separate animal type, assay-type titre magnitude terms, and titre measurement units effect. Other, more complicated models such as the fold drop models which constituted the main model of the paper also employed dataset-dependent slope terms to account for differences in fold drops between data coming from different labs, which we did not observe in the raw data here. The simplest model mentioned in [23] is the most similar one, where only an independent dataset titre magnitude effect is used, and it differs from the model used here by the fact that we employ a hierarchical model in which dataset titre magnitude effects depend on the estimated assay-type titre magnitude effects. We employed this approach because titre magnitude between labs using even the same assay type seemed too variable to be modelled by a single assay-dependent term, and yet we also wanted to obtain estimates of how assay type affects titre magnitude and how variable it is, which would not be possible using independent dataset titre magnitude terms.

The Bayesian model described above is given by

$$L\left(D_{ij}|t,\alpha ,\sigma ,\nu \right)∼CST\left(D_{ij}|t_{i}+\alpha_{j},\sigma_{i},\nu \right)$$

$$p\left(t_{i}\right)∼N\left(t_{i}|t_{i}^{0},1\right)$$

$$p\left(\alpha_{j}|\mu_{a\left(j\right)}\right)∼N\left(\alpha_{j}|\mu_{a\left(j\right)},2\right)$$

$$p\left(\mu_{a}\right)∼N\left(\mu_{a}|\mu_{a}^{0},1\right)$$

$$p\left(\sigma_{i}\right)∼\gamma^{-1}\left(\sigma_{i}|1,0.5\right)$$

$$p\left(\nu \right)∼\gamma^{-1}\left(\nu |5,2\right)$$

Likelihood of observing the data D_ij_ given the parameters. Prior probabilities: Mean titer of variant i; Bias of dataset j; Bias assay type a; Bias SD of assay type a; SD of measurement error.

where D_ij_ is the log titre observed for antigen i by lab j, t_i_ is the titre of antigen i against the serum NIBSC 21/338, α_j_ is the titre magnitude for dataset j, μ_a_ is the titre magnitude for assay type a, σ_i_ is the per antigen measurement error, and ***ν*** is the degrees of freedom. Note that titre magnitude for dataset j is estimated as a normal distribution with mean given by the assay-type titre magnitude and standard deviation given by assay-type offset standard deviation (i.e., a hierarchical model). CST(x|μ, σ, ***ν***) is the censored StudentT likelihood probability distribution function, which uses the StudentT probability density when observation D_ij_ is not censored and uses the StudentT cumulative probability density when it is. N(x|μ, σ) is the normal distribution probability density with mean μ, and sd σ and Ɣ^−1^(x|μ, σ) is the inverse gamma probability density (expressed in terms of mean and scale parametrisation). Below, we also test StudentT against a normal likelihood model, in which case ***ν*** is not used. t_i_^0^ and μ_a_^0^ are, respectively, non-parametric point estimates for geometric mean titre and assay biases. See the next paragraph for more about this formulation. We sampled this model in PyMC with the following parameters: tune = 2000, draws = 5000, chains = 6.

Note that if the priors for t_i_ and α_j_ were completely flat, the model would be unidentifiable (since they appear as a sum in the likelihood that adding a constant to one of them and subtracting from the other gives the same likelihood). We therefore choose priors centred around point estimates but with standard deviations comparable to a natural unit of scale for the problem at hand (the standard deviation of data mean centred at zero is roughly one). Even though the priors circumvent this problem to some extent, it is still preferable in such cases to report centred observables which are agnostic to this identifiability problem. We employ the following centralisations when producing the posterior distributions: α_j_ ← α_j_−E(α), μ_a_ ← μ_a_−E(μ), and t_i_ ← t_i_ + E(α), where E(α) represents the mean of titre magnitudes across labs and E(μ) represents the mean of titre magnitudes across assays. These centralised observables are used when making the figures in this manuscript.

#### 2.5.2. Modelling Landscapes

In this manuscript, we present for the first time a purely Bayesian approach to landscape modelling that also takes into account thresholded titres using censored distributions. Correct handling of thresholded titres is essential when the dataset contains escape variants like XBB.1.5, which might have abundant thresholded titres. The first application of titre landscapes was presented in [41], where landscapes were fitted to individual HI titre data using local linear regression around each antigen and a linear weighing function was used to deal with fold drops involving thresholded titres. A maximum likelihood approach taking into account censoring was then employed in [42]. In this case, we fit a single landscape to a group of titres where groups are determined by Bayesian clustering (see next section). Instead of local linear regression, we use geometric sum of multiple cone-shaped landscapes (we use model testing to determine optimal number of cones), rather than local linear regression. Local linear regression is more suitable for cases when the exposure history is more complicated. Cone-shaped landscapes are more suitable when, for instance, convalescent sera from previously naive animals or humans relatively early on in a pandemic are used. And instead of a maximum likelihood approach, we use a Bayesian approach, which not only allows us to use the prior knowledge of antigen positions more effectively but also to quantify the uncertainty in landscape coverage. In [43], cone-shaped landscapes are used also to fit sera from individuals after bivalent boosting with WT + BA.1 or WT + BA.4/5 using the package in [42]. We preferred to formulate this model first of all to be able to test multiple cones (which is not available in [42]) and to also have a single Bayesian framework so that we can have HDI estimates for the landscapes coverage (see Figure S20).

In this section, index i indicates antigens and index j indicates sera in a chosen group. We also assume that there exists a map of antigens in which c_i_ represents the 2D coordinates of the antigen i. The landscape is modelled as a combination of one or several cones over this map (combination is defined by a function F, which is explained below).

$$L\left(D_{ij}|\beta ,h,\sigma ,\mu ,\xi ,\nu \right)∼CST\left(D_{ij}|F\left(h_{s}-\beta_{s}\right.\right.\left.\left.\times d\left(\mu_{s},c_{i}\right)+\xi_{j}\right),\sigma ,\nu \right)$$

$$p\left(\mu_{s}\right)∼MN\left(w_{\mu },\mu_{0},0.25\right)$$

$$p\left(h_{s}\right)∼MN\left(w_{h},h_{0},0.25\right)$$

$$p\left(\beta_{s}\right)∼\gamma^{-1}\left(0.8,0.25\right)$$

$$p\left(\xi_{j}\right)∼N\left(0,0.25\right)$$

$$p\left(\sigma \right)∼\gamma^{-1}\left(0.75,0.25\right)$$

$$p\left(\nu \right)∼\gamma^{-1}\left(10,2\right)$$

Likelihood of observing the data D_ij_ given the parameters. Prior probabilities: Apex coordinates of cone s; Height of cone s; Slope of cone s; Bias of serum j; SD of measurement error; Degrees of freedom.

Here, D_ij_ is the log titre of antigen i against serum j, **d** represents the Euclidean distance, μ_s_ is the apex (two-dimensional vector) of cone s, **h_s_** is the height (real) of cone s, and **ꞵ_s_** is the slope (real) of cone s. **MN**(**w, μ, σ**) is a mixture of normals with weights w, centres μ, and sd σ. w, μ, σ are basically chosen such that the “sampled cones” have their apexes chosen from the nearby regions of available antigens in the map and similarly heights from the mean titre value for these antigens (see Determining the Optimal Number of Cones Section in SI for further discussion of this). Therefore mixture priors add sufficient flexibility to the model without overfitting (if the centres are restricted as described above). **ξ_j_** is the per serum bias, and σ is the measurement standard deviation. CN and N are as in the previous section, and **Ɣ^−1^**(**μ, σ**) is the inverse gamma probability density in location and scale parameterisation. **CST**(**x**|**μ, σ, ***ν*****) is the censored StudentT likelihood. We normalise the data so that each serum is centred around the gmt of the data, hence the reason why the bias prior has relatively small standard deviation. We sample this model in PyMC with the following parameters: tune = 2000, draws = 4000, chains: 6.

The function F that appears in the likelihood operates over the index s of the cones (if there are multiple) and combines them into a landscape. In principle, there are various ways to combine the cones into a single landscape, but we have used geometric sum. Therefore, for instance, for two cones, one would have F(x, y) = x + y. Validating the correct combination method would probably require more antigens and multi-exposure sera with precisely known history. This was, for instance, applied to multi-exposure ferret reinfection sera and geometric sum was seen to be the most efficient (personal communication D. J. Smith, work in progress).

The fixed inputs to the apex centre and height mixtures can be determined by directly looking at the available data. Note that the number of the components of the mixture is independent from the number of cones. One simple way to construct the apex centre mixture is to take a couple of antigens which seem most likely to be local maxima from data and similarly compute the height mixture centres from the mean of the log titres at these antigens.

Note that in general, mixture models can suffer from non-uniqueness (such as label-mixing problems) and multi-modality of solutions. These problems especially manifest themselves when one wants to perform statistics on parameters involved in the mixture, such as μ_s_**.** However, the problem is remedied (provided other diagnostics such as chain convergence, etc., are fine) if one is interested in observables agnostic to labels. In this case, we are interested in the observable F(h_s_ − d(μ_s_, c_i_) × ꞵ_s_ + ξ_j_), which is the combination of different cones (over index s) and therefore agnostic to order of label s. Nevertheless, we employ some tricks of the trade for working with mixture models such as ordering of the x coordinates. Combined with a somewhat informed prior, one may still be able to perform statistics on quantities such as μ_s_ without problems if their posterior distributions look unimodal. On the other hand, if one is simply interested in visualisation of individual cones, then sampling an observable where F is replaced by maximum will also produce a landscape agnostic to this.

#### 2.5.3. Clustering Titres

In this preliminary study, the available metadata are generally coarse and can only be used to provide a lower bound on the number of infections and vaccinations (with sometimes unidentified variants). Information regarding time since infection/vaccination are commonly not available. We provided a breakdown of the data where grouping is carried out by metadata in the SI, Figure S22. Here, we followed an unsupervised clustering approach instead. We collated the in-house human serum data from all the labs which have provided data for the control serum NIBSC 21/338 and which have titrations against four or more antigens (AHRI, Emory, Innsbruck, FIVI, HKU, VIDRL NICD, and Genève). We then clustered these data (sera with all thresholded titres were not included) using a Bayesian Mixture of Normals approach (see SI, Mathematical Methods). There are multiple reasons why we employ Bayesian clustering. The main reason is that out-of-the-box methods such as Euclidean k-means clustering or classical Gaussian mixture models do not allow modelling censored data. It is evident that there are ample censored titres in this dataset, and therefore not modelling them appropriately could lead to erroneous cluster assignments. A second benefit is that Bayesian modelling allows one to quantify probability of each serum belonging to a group, and therefore it is possible to draw a meaningful threshold in which sera which have high probability of belonging to two groups (such as 0.6 vs. 0.4) can be discarded as uncertain. The third benefit is that one is able to relatively easily add a separate cluster for outliers [44]; therefore, one can also discard sera that do not really resemble any of the non-outlier groups. The major drawback is the problem of non-uniqueness and multi-modality. Label mixing is a technical difficulty that would in this case make the interpretation of the results meaningless, whereas multi-modality is a more fundamental issue which could suggest that chosen parameters (such as number of clusters) are too flexible or too stringent. Both problems could manifest the same symptoms such as multi-modal posteriors but for different reasons. Using a transformed coordinate system coupled with ordering on the first coordinate, using priors centred on pre-computed solutions such as Euclidean means (but still giving them enough flexibility for exploring different solutions), and including a fixed outliers cluster seems to remedy this problem and allows us to reach up to five sufficiently well-defined and distinct clusters within a dataset of 158 sera alongside an outliers cluster that indeed seems to pick up sera that do not resemble any of the other groups. We also employ diagnostic criteria such as a Bayesian version of the classical silhouette plot, which seems to correctly identify when clustering goes wrong.

While fitting the clusters, the data are mean centred since absolute magnitude of titres is irrelevant for this purpose and we are more interested in relative differences between titres. In order to deal with groups of sera which do not have a full set of measurements (contrary to experimental design, many labs have titrated against a limited set of antigens for various reasons), we first group the data into patterns of missing data (for instance all those data which are missing the measurement against the first antigen for one group, etc). The likelihood in the model below is applied to each group separately and only on antigens for which the group has no missing data.

$$L\left(D_{ij}|w,mu,n,\sigma \right)∼MN\left(D_{ij}|w,\mu +b,\sigma \right)$$

$$p\left(w\right)∼D\left(\alpha \right)$$

$$p\left(\mu_{k}\right)∼N\left(\mu_{k}^{0},1\right)$$

$$p\left(b_{j}\right)∼N\left(0,0.5\right)$$

$$p\left(\sigma \right)∼\gamma^{-1}\left(0.5,0.25\right)$$

Likelihood of observing the data D_ij_ given the parameters. Prior probabilities: Mean cluster weights; Center of cluster k; Bias of serum j; measurement error.

Here, **w** is the mixture weights, and **ɑ** is the concentration parameter of the Dirichlet distribution **D**. **μ_k_** is the centre of cluster k (a parameter vector of dimensions equal to number of antigens), and **μ^0^_k_** is the centre (a fixed vector of dimensions equal to number of antigens). **b_j_** is the bias of serum j, and **σ** is sd for the mixture of normal likelihood. We sample this model using PyMC with the following parameters: tune = 2000, draws = 2000, chains = 4, target_accept = 0.9, and init = advi + adapt_diag.

If number of clusters > 1, we extend μ_k_ by a single set of fixed coordinates representing the outliers with a large sd. An easy choice for μ_outlier_ is the mean of the data and scalar multiple (2–4 seems to suffice) of the mean standard deviation of the data. Since mixture models already suffer multi-modality, it is probably best to estimate the outlier cluster variables non-parametrically as such. A more refined choice is for μ_outlier_, and its sd could be the μ_k_ and a multiple of σ obtained from fitting a single cluster to the data (which is what we have followed with 4σ as the sd).

**ɑ** is chosen as in [20, …, 20, 2], where last entry represents the weights of the outlier cluster and others the non-outlier clusters. This is meant to kindly discourage small non-outlier clusters and very large outlier clusters. The fixed centres **μ^0^_k_** are derived from k-means solutions (in case of missing data, we apply an iterated k-means scheme until convergence, see code repo [40]). Even though the solutions obtained from k-means will no doubt affect the solutions of the Bayesian model (especially the smaller groups), the standard deviations for centre coordinates are left relatively vague (compared to a mean data standard deviation of about 1.2 after mean centring) and therefore can easily explore alternative solutions or correct biases due to Euclidean k-means not taking into account censored titres. In the Clustering Diagnostics Section in the SI, we present additional analysis which demonstrates that the Bayesian clustering method can handle titre curves with many thresholded titres more correctly than Euclidean k-means.

Finally, as mentioned before, we actually use a transformed coordinate system. Assuming that each antigen gives a single coordinate for the serum measurement, we have six coordinates in this problem. We use the transformed coordinate system

$$T(x_{1},\;x_{2},\;x_{3},x_{4},\;x_{5},\;x_{6})\;=\;(x_{1}+x_{2}+x_{3}-x_{4}-x_{5}-x_{6},\;x_{2},\;x_{3},\;x_{4},\;x_{5},\;x_{6})$$

The only change is in the first coordinate, which basically replaces the first titre by a difference of the first three titres and last three titres (and σ for this coordinate is, respectively, scaled by sqrt(6)). Moreover, an ordering transformation is applied to this coordinate (which means that given n > 1 clusters, the first coordinate T_1_ of the transformed cluster centres will always be ordered). This transformed and ordered choice of coordinates is meant to distinguish data based on their difference of titres between earlier and later variants and therefore prevent label mixing—for sufficiently low number of clusters. We call the first coordinate T_1_ the rank. Even though mixing can occur for a large number of clusters, we can at that point argue that the number of clusters represents a resolution finer than required.

Finally even though the model is set up so that it uses an independent set of univariate normals for the cluster centres, from the perspective of PyMC, such priors are very liable to mixing (basically, random cluster centre sampling occurs not just between clusters but also along coordinates). A remedy is using multivariate normals as is normally undertaken in clustering; however, one cannot define censoring for multivariate distributions in PyMC. Another option is defining labels for data explicitly and using the automatic marginalisation feature of PyMC experimental, but this experimental feature does not allow one to develop complex models where non-measured data can be handled. So we developed a workaround in which a set of independent univariate censored normals is wrapped as a single multivariate distribution (therefore censoring first and then turning it into a multivariate distribution) so that coordinates for a cluster are sampled together. For mathematical purposes, this is identical to a set of independent univariate censored normals, but it samples more efficiently.

In order to measure the efficiency of clustering, we define a Bayesian version of the silhouette plot. A silhouette plot in the classical setting measures roughly how well on average data belong to their cluster (by looking at the relative difference between how close a datum is to its cluster vs. how close it is to other clusters, i.e., relative cohesion—separation). There is a much more natural construction of that in the Bayesian world. Let p_ij_ be the probability that point i belongs to cluster j. Then cohesion of point i is max_l_ p_ij_ and separation of point i is sum_j_ p_ij_-cohesion_i_. Then the silhouette score of a point is cohesion_i_-separation_i_. When performing further analysis, such as building landscapes, one can also clean groups by putting a lower threshold on the probability of a point belonging to its cluster. Lower values can be interpreted as a point which could potentially be swayed easily to another cluster by measurement noise and therefore its cluster cannot be identified reliably. When defining members of the group, such as in Figure 4A, we have only taken sera for which cohesion was less than 0.1.

## 3. Results

### 3.1. Comparability of the Neutralisation Readout Across Laboratories and Across Assays

A total of 15 laboratories successfully conducted experimental work for comparability analysis (see Table S1 for a summary of each lab’s experimental setup). Of those, two laboratories extended the analysis to also use comparable viral strains that were independently isolated (termed “in-house” in the figures). One laboratory enrolled in this study and provided data but did not participate in the comparability analysis. Four types of titre measurement methods were tested in total: Microneutralisation Titres (MicroNeut, n = 5), Plaque Reduction Neutralisation Titres (PRNT, n = 7), Focus Reduction Neutralisation Titres (FRNT, n = 6), and Pseudovirus Neutralisation Titres (PseudoVirusNeut, n = 2, Lentivirus). All three except PseudoVirusNeut are live virus assays. The sequences for these isolates and the isolates sent to the labs can be found in the GitHub repo [40], and the accession numbers can be found in Table S2 (see also Figure S24 for a sequence plot).

### 3.2. Geometric Mean Titres

The raw titres against the positive plasma pool (WHO International Standard 1st International Standard for antibodies to SARS-CoV-2 variants of concern NIBSC code: 21/338) for each of the labs (including in-house virus isolates from Emory and Innsbruck laboratories) are displayed in Figure 1. The negative pre-pandemic control plasma pool (20/142) was below detection limit (lowest titre tested) across all labs and assays. For each variant tested, the relative differences in titre magnitudes for each variant were largely comparable across assays (PRNT, FRNT, MicroNeut, and PseudoVirusNeut) and laboratories with the only exception of titres generated by RLID for Alpha, Beta, and Delta variants using the PRNT assay (Figure 1A). Given the overall compatibility of these data in fold drops (RMSD between the mean centred GMT and measured titres was 0.56), we used a model which estimates these titres as a sum of two sets of variables: 1. A geometric mean titre (GMT) value for each antigen (6 parameters) and 2. A hierarchical overall titre magnitude value (with the parameter name offset) for every lab (20 parameters) controlling for the overall magnitude of the titre, which depends on the estimated titre for each assay platform (4 parameters). Precise definitions of these parameters are given in the SI in the Modelling Assay-wise Effects and GMTs Section. However for practical purposes, dataset titre magnitude (or offset for short) can be thought of as the difference between titre means (over antigens) between the estimated GMTs and lab titres. It is a relative measure of how high on average titres from one lab are compared to others (see also Assay Offsets Section). The GMT estimates from all laboratories and assays align very well with the lab raw data once the titre magnitudes are controlled for with the exception of the GMT estimates for Alpha, Beta, and Delta generated by RLID (Figure 1B, RMSD: 0.59, see also Model Diagnostics Section for further analysis of the goodness of fit and posterior predictive checks and Figure S5 for a pairwise comparison of fold drops). Excluding RLID and in-house samples, the per antigen RMSD of titre magnitude-adjusted lab titres to the GMT line were as follows: Alpha: 0.39, Beta: 0.44, Delta: 0.87, BA.1: 0.28, BA.5: 0.64, XBB.1.5: 0.72 (calculated via the posterior means). The fitted per antigen standard deviation posteriors had means as follows: Alpha: 0.53, Beta: 0.54, Delta: 0.68, BA.1: 0.67, BA.5: 0.81, XBB.1.5: 0.71 (without excluding RLID and in-house samples).

### 3.3. Assay Offsets

**Figure 2:**
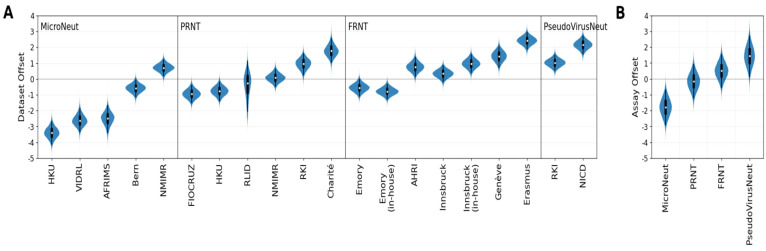
Posterior distributions for lab-wise and assay-wise offset means. (**A**) This shows the posterior distribution for the offset parameter for each lab where each lab’s offset is a normal distribution whose mean is given by the assay-type offset whose posterior distributions are shown. (**B**) The white marker shows the mean, the thin vertical black line shows the 94% HDI, and the thick vertical black line shows the interquartile range.

In the hierarchical model used, each lab’s overall titre magnitude is a parameter that is drawn from a normal distribution with mean equal to the titre magnitude estimated for that lab’s assay type (MN, FRNT, PRNT, or PseudoVirusNeut). The former parameters are called dataset offsets and the latter assay offsets. By looking at the distribution of estimated offsets, there was a general trend for Microneutralisation titres to be lower and Pseudovirus Neutralisation Titres to be higher than titres measured by FRNT or PRNT (Figure 2B). Sampling the differences of offsets for each assay type to Microneutralisation reveals that with a 94% HDI for Pseudovirus Neutralisation’s offset difference to MicroNeut is significantly positive (Figure S7). However, we did observe large variability for dataset offsets within the same assay group (Figure 2A). The 94% HDI bars for pairwise difference in assay offsets were observed to span three to four units (Figure S7). It is likely that the offsets depend on other parameters such as the method of computation of titres; hence, without controlling such variables, a finer conclusion cannot be presented.

### 3.4. Fold Drop

**Figure 3:**
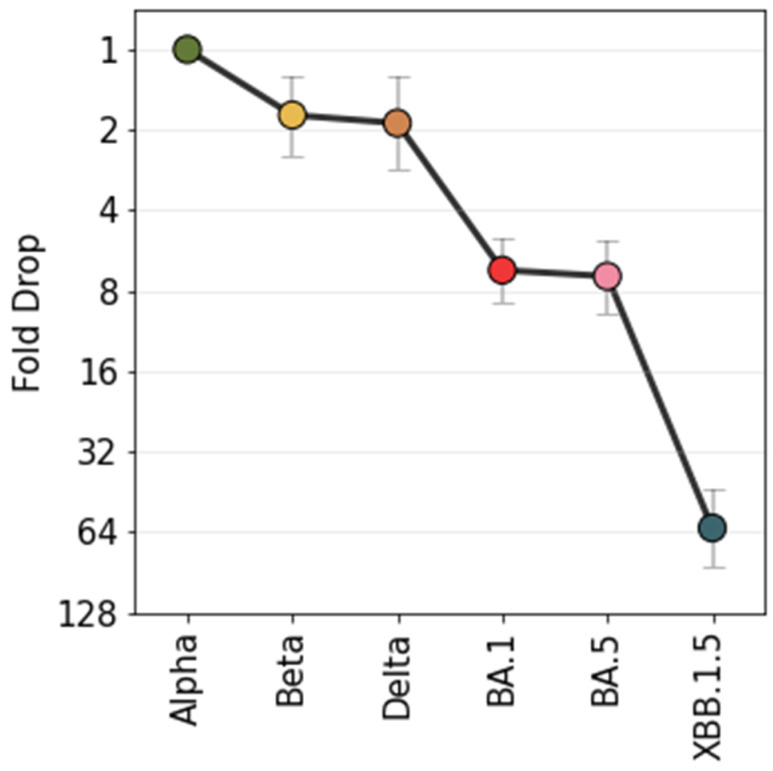
Fold-change reduction compared to Alpha. The figure shows the estimated fold drops of variant titres compared to Alpha. The bars indicate the 94% HDI for the posterior of estimated fold drops.

The fold drop in neutralisation between different variants tested against the IS (pooled control serum NIBSC 21/338) represents the antigenic distance between the variants regardless of titre magnitude. We observed an almost 64 mean fold drop (42.3–81.1 94% HDI) of XBB.1.5 titres vs. Alpha as opposed to a 1.7 mean fold drop for Beta and Delta (1.2–2.3 and 1.2–2.6 94% HDI, respectively) and a 6.5 mean fold drop for BA.1 and BA.5 (4.8–8.2 and 4.8–9.0 94% HDI, respectively). These fold drops are shown in Figure 3. Fold drops with respect to other antigens and the respective high-density intervals are shown in Figure S6.

### 3.5. An Initial Step Towards Global Immunological Surveillance

Population immunity landscapes, which refer to the spatial and temporal distribution of immunity within a population, are useful tools for evaluation of vaccine update strategies both prospectively and retrospectively [41]. Landscapes build “titre surfaces” on a two-dimensional map that represents the antigenic relation between variants as opposed to titre curves on a one-dimensional ordinal axis of antigens. They greatly facilitate interpreting relationships between sera that have been titrated against many antigenically distinct variants. This section highlights how to build landscapes from multiple labs’ sera archives using a Bayesian approach. The metadata available for the sera are coarse, and therefore, we apply unsupervised grouping methods to extract patterns out of the data before constructing the landscapes.

We analysed data provided by a subset of the labs (AHRI, Emory, Innsbruck, FIVI, HKU, VIDRL NICD, Genève, and FIOCRUZ) where WHO stock viruses were titrated against their in-house human sera (N = 268). We analysed the data by grouping similar sera together via a Mixture of Censored Normals Model (within a Bayesian framework) and then fitted landscapes to each group (see the sections Clustering Titres and Modelling Landscapes in the SI for details). More than half of the serum set comes from individuals multiply vaccinated with WT vaccines (sometimes bivalent) and no reported/known infections (N = 151). The characteristics of the remaining sera are quite variable and, for instance, contain sera from individuals with confirmed BA.1, BA.5, XBB.1.5 infections (sometimes with no reported vaccinations and no other infections and sometimes with multiple infections) and many sera with unidentified variant infections and sometimes unspecified bivalent boosters. The full dataset with the available metadata is available in [40]. A table which shows a breakdown of the sera is given in Table S3.

As the main result of this analysis, we see that the largest cluster that corresponds to roughly 80% of the data is clustered in a group whose mean fold-change drops look very similar to IS (NIBSC 21/338 pooled plasma) (Figure S15). This is not surprising because the metadata available suggest that these sera come from individuals who were likely vaccinated/infected multiple times with index virus and/or early VOCs, much like the constituents of the IS. It is also seen that non-parametric point estimation of fold drops for this group underestimates fold drop of XBB.1.5 by about two-fold (Figure S15).

**Figure 4:**
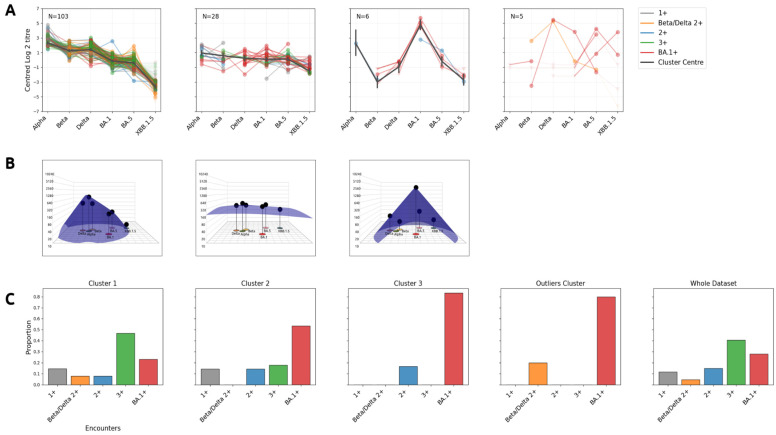
The cluster groups, landscapes, and metadata. (**A**) The plot shows the three non-outlier clusters and the outlier cluster obtained from clustering the labs’ collated serum data. The black line shows the (mean centred) representative titre of the cluster, and the bars indicate the 94% HDI for the estimate. The coloured lines indicate individual serum titres after each serum’s titre magnitude (with respect to black line) was subtracted. Lower and upper triangles indicate titres censored from below and above. Colouring of the sera indicates the type of encounter, and encounter types’ colours are given in the legend. n+ indicates that the serum had encounters of infection and vaccination (at least n different times) with Alpha, earlier than Alpha, or unknown variants. Beta/Delta 2+ means that the serum had at least two encounters, one of which was confirmed Beta or Delta. BA.1+ indicates that the serum had at least one encounter with a BA.1 or later variant. The markers (and line segments left of these markers) for thresholded titres are shown in a more transparent colour. This figure shows only the sera whose probability of belonging to any other cluster is less than 0.1, see Figure S14 for others. (**B**) This shows the landscapes fitted to the non-outlier clusters of sera shown in (**A**). The base map is from [23]. There is no unique lower limit of detection for the data since different labs have different ranges of dilutions. However they are all generally close to 10 (after the bias estimated in the first section is removed); therefore, in this figure, the lower limit of detection is also set to 10, which is shown by the base plane. One landscape per group is fitted due to the low number of antigens involved in this study. Interactive html plots can be found in the code repo [40]. (**C**) Bar plots indicate the distribution of encounters (infection or vaccination) of the sera for each cluster (as shown in (**B**)) as well as the overall distribution for the whole dataset (last bar plot). See Figure S13 for further breakdown of these categories.

The second pattern looks more cross-reactive and comes from a set of individuals in which the proportion of BA.1 or later variant exposures to those with only index infection/vaccination is much higher compared to the first group (Figure 4C and Figure S13). The metadata available suggest that these encounters are BA.1, BA.2, BA.5, Delta + BA.1, XBB.1.5, and some unidentified variants. Looking at the landscapes (Figure 4B and Figure S20), this is the only group that consistently covers XBB.1.5 well beyond its full 94% HDI range, whereas the other two groups cover only up to BA.5 or up to XBB.1.5 at their border (which is susceptible to change if the threshold plane’s position changes by a unit or HDI is increased). Finally, the third pattern picked up by clustering comes from a set of individuals who are known to be BA.1-convalescent (without vaccination) plus one individual with two unidentified infections and no recorded vaccinations. The outlier cluster contains sera like BA.1, BA.5-convalescent individuals, or individuals with Delta, XBB.1.5 infections, and a WT vaccination.

The fact that the first group contains sera from uninfected individuals who were involved in a trial with BA.1 + index or BA.5 + index bivalent vaccine is likely an effect of imprinting due to earlier multiple index vaccines [45,46]. Indeed, the second, more cross-reactive group involves many individuals who are non-vaccinated and convalescent with BA.1, BA.2, and XBB.1.5 as well as those convalescent with XBB.1.5 and vaccinated with index. The proportion of no reported infections and multiply index-vaccinated individuals is much smaller compared to the first group (compare cluster 1 vs. cluster 2 in Figure S20). A similar difference between only 2×/3× index-vaccinated and index-vaccinated + BA.1-infected individuals was seen in [47]. However, it is hard to make more detailed and definitive statements due to the uncertain nature of the metadata. A further breakdown of these sera by metadata is given in Figure S13, and details about the process of clustering are given in the section Clustering Diagnostics.

## 4. Discussion

The findings of this study demonstrate a high degree of compatibility in SARS-CoV-2 neutralisation assay results across multiple laboratories, with minor variations observed. This suggests that despite differences in methodologies and laboratory settings, titres measured by different labs using different assay types can be compared, especially when adjusting for different assay/lab titre magnitudes. The lab/assay-specific titre magnitudes may be determined using the International Standard. The Bayesian model employed for estimating titres effectively accounted for laboratory-specific mean titre magnitudes, revealing a stark alignment of results with the overall trend. This study also suggests that the use of the same virus isolate across laboratories is not a necessity, but sequence harmonisation might be sufficient; pseudoviruses generated for the same sequences and to some extent live virus variants available in-house (with some sequence differences) yielded comparable results to WHO stocks (see Figure S5). This will greatly reduce the logistical and financial requirements for future assessments.

The differences that were observed are most likely attributable to one or more of the following: viral isolate propagation methods, choice of cell lines, and assay protocols. For example, while we do not judge that the difference in Alpha, Beta, and Delta titres generated by RLID is due to cell type, as this lab is not the only one that has used Vero E6 cells, there is nevertheless accumulating evidence that the choice of cell lines has an impact on assay results [48]. Another interesting difference is seen in the titres against the Delta variant for Innsbruck between the shared and the in-house isolates (see Figure S5). The spike protein differences were A771S, G1167 (WHO has G1167V), and G1219 (WHO has G1219V) (see Table S2 and Figure S24). This may be due to the fact that the in-house isolate was an early Delta isolate (B.1.617.2) as opposed to the shared one, which was a late Delta isolate (AY.43). Considering convergent evolution in the SARS-CoV-2 spike, this could explain the higher fold drop for the in-house virus isolate. Additionally, differences in assay sensitivities and thresholds may impact the detection of neutralising antibodies, highlighting the importance of standardised procedures and quality control measures. Incorporating censored modelling approaches, as demonstrated here, can also remedy this problem to some extent.

The study findings have significant implications for vaccine development, as they provide valuable insights into the likely effectiveness of existing vaccines against emerging SARS-CoV-2 variants. The data shown here confirm the immune escape of the assessed XBB.1.5 variant in sera from people exposed only to pre-Omicron variants as previously published [49] as well as the estimated GMT for the viral isolates provided in this study. Therefore, setting up a systematic and comparable methodology to determine the immune escape capabilities of variants is crucial for optimising vaccine formulations and guiding vaccination strategies. By assessing neutralisation titres against key variants, researchers can inform decisions regarding the need for vaccine updates or booster doses. Furthermore, the compatibility of assay results across laboratories underscores the reliability of global surveillance efforts in monitoring the impact of new variants on immune escape.

While this was not the goal of this study, the rationale for the WHO to support harmonising antigenic characterisation data is, just like for influenza, to identify quantitative thresholds for neutralisation assays that can be used to support decision-making processes about vaccine antigen composition. For example, the 8-fold drop measured between BA.1/BA.5 and XBB.1.5 in this study would likely warrant considering a change in vaccine antigen composition. On the other hand, the drop measured between Delta and Omicron was 4-fold, which is not as high as the drop measured by other studies that used sera of vaccinated individuals (from 6 to 25 fold drops) [50]. When using sera from infected and vaccinated individuals, more relevant to this study, the fold drop between Beta and Omicron was 3–4-fold [51], which is comparable to what is found in this study. However, there was significant variability across studies, almost certainly reflecting the differences in underlying population immunity of the sera tested. This may be due to the fact that the WHO International Standard 21/338 was created as a pool of many individuals specifically to have a wider breath of responses than single individuals. This reference preparation was not meant to be representative of any population immunity, as the intended use is as the primary calibrant to harmonise results from assays from different laboratories using the same viral isolates. Regardless, neutralisation of the emerging variant using human (community) sera is not the only set of critical laboratory data that are needed to consider changes in vaccine antigen composition. For example, anti-sera (i.e., sera of naive animals then infected with the emerging variant under consideration for vaccine change) provide a more precise measure on how immunogenic this variant may be (and therefore how well the new vaccine would cross-protect against other variants). These are also very critical data, as some variants may be less immunogenic than others [52]. The recent establishment of the WHO Coronavirus Network (CoViNet) will hopefully work as a platform to establish such thresholds [53].

This study has several limitations. Firstly, logistics has been time-consuming, as sharing virus isolates and the International Standard took time, and some laboratories experienced delays in receiving the material. Also, some laboratories were not able to rapidly propagate enough virus material. Therefore, and considering the results we presented, a way forward could be that, whenever feasible, laboratories generate their own isolates and the viral sequence is checked after each passage. Secondly, a hamster map from another source was used in the human serum landscape analysis. Although for the antigenic characterisation of influenza virus it is common to use maps generated using ferret sera, for SARS-CoV-2, we are still in the exploratory stages of comparing the behaviour of human versus hamster sera. Finally, human serum metadata were relatively poor in this study, and global surveillance of SARS-CoV-2 antigenic characterisation would certainly benefit from more detailed metadata.

## 5. Conclusions

Continued research in global immunological surveillance is essential for building population immunity landscapes and guiding vaccine update strategies prospectively and retrospectively. Further investigations into the factors influencing assay variability, such as cell line choice and assay sensitivities, are warranted to enhance assay standardisation and comparability. Additionally, ongoing monitoring of SARS-CoV-2 variants and their immune escape capabilities is critical for staying ahead of the evolving threat posed by the virus. In conclusion, the comprehensive analysis of neutralisation assays conducted across multiple laboratories provides valuable insights into immune responses against SARS-CoV-2 variants. The high degree of assay comparability observed underscores the reliability of global surveillance efforts and emphasises the importance of continued vigilance and adaptive public health responses. It also suggests that virus sharing may not be always needed if the key mutations—as per in silico prediction—of the emerging variant under study are present in “in-house” viral isolates. By understanding the dynamics of viral evolution, immune escape, and vaccine effectiveness, researchers can better address the challenges posed by SARS-CoV-2 and its variants.

## Data Availability

All the titrations generated by the labs against the WHO International Standard as well as their own sera (where available) against WHO BioHub variants and in-house variants (where available), the GISAID sequences for the variants that are used in the study, the metadata available for each of the labs’ own sera, and the models used for the analysis are available freely in the GitHub repository [40].

## Supplementary Materials

The following supporting information can be downloaded at: https://www.mdpi.com/article/10.3390/v16121936/s1, Figure S1: Posterior and trace plots for GMT, offset mean and SD; Figure S2: Posterior predictive and Loo PIT; Figure S3: Pareto-k values for different models with and without RLID excluded; Figure S4: Comparison of StudentT and normal models; Figure S5: Comparison of fold drops between each lab and fitted GMT; Figure S6: Fold drops of titres with respect to each variant; Figure S7: Pairwise difference between assay-type offsets; Figure S8: Likelihood and silhouette scores for different numbers of clusters; Figure S9: Differences between cluster centre fold drops for four and five clusters; Figure S10: Differences between cluster centre fold drops for three clusters; Figure S11: Posterior distributions for cluster centres and rank, trace plot for rank; Figure S12: Clusters obtained with Euclidean k-means; Figure S13: Serum encounter categories of clusters; Figure S15: Comparison of fold drops obtained via various methods; Figure S16: Posterior predictive and LOO PIT plots for the landscapes; Figure S17: Posterior predictive and LOO PIT plots for the landscapes for the first group split into antigens; Figure S19: Model comparison for different number of cones; Figure S20: Landscapes fitted to three groups and their HDI; Figure S21: The antigenic map used for landscapes; Figure S22: The raw titres for human sera obtained from labs; Figure S23: Laboratory enrolment process and geographical representation; Figure S24: Comparison of spike sequences for the variants used in this study; Table S1: The assay information for the labs involved in this study; Table S2: The accession numbers for sequences of variants used in this study; Table S3: The infection and vaccination categories of sera used in this study.
