# Supplementary material for: A Global Collaborative Comparison of SARS-CoV-2 Antigenicity Across 15 Laboratories
Source: Viruses. 2024 Dec 18;16(12):1936. doi: 10.3390/v16121936 (PMC11680265; doi:10.3390/v16121936)
Supplement: Supplementary file 1 [file viruses-16-01936-s001.zip › viruses-3338785-supplementary.pdf]

Model Diagnostics & Additional Analysis. This section details diagnostics (convergence and posterior checks) for the three models used as well as figures for some extra analysis mentioned but not presented in the main text.

Fitting Diagnostics for GMT and Offset Model. The posterior and trace plots in Fig.S1 and Fig.S2 indicate good convergence and the posterior predictive plot indicates a good fit.

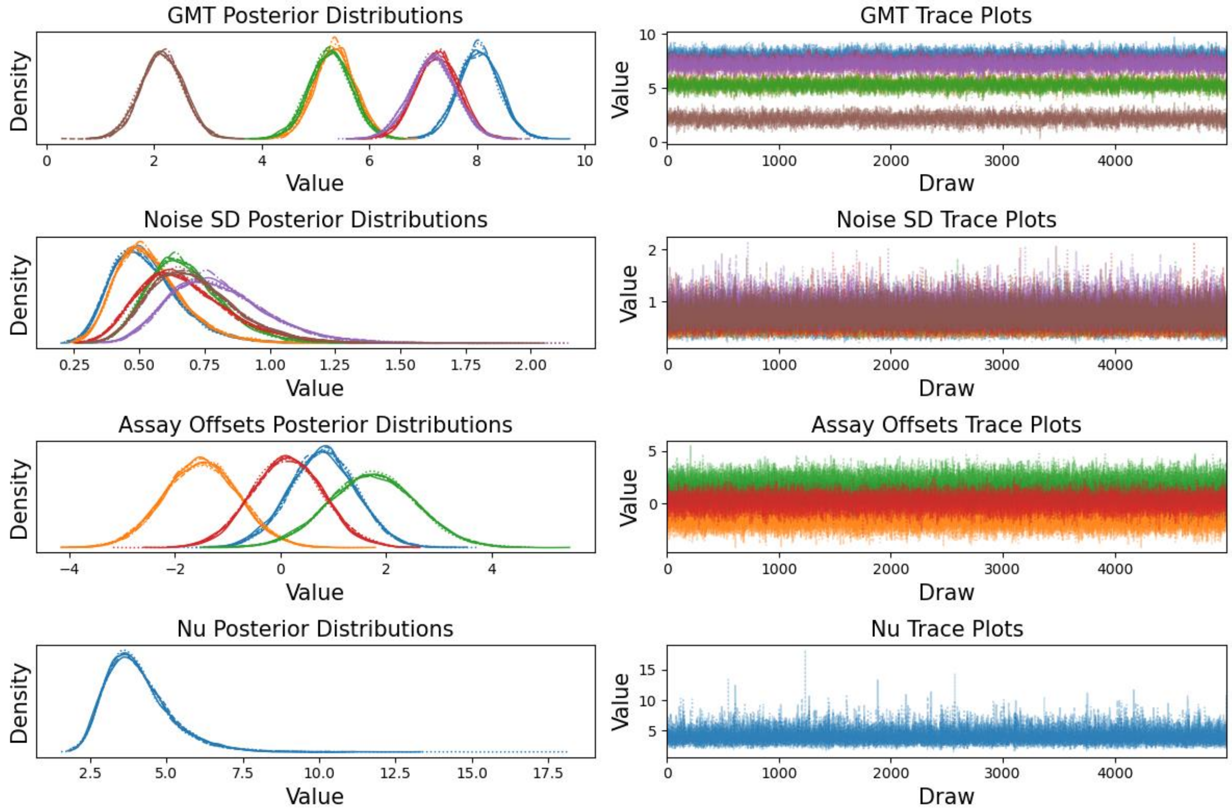

Figure S1: Posterior and Trace Plots for GMT, offset mean and SD. Each row shows the posterior and trace plot for the variables indicated.

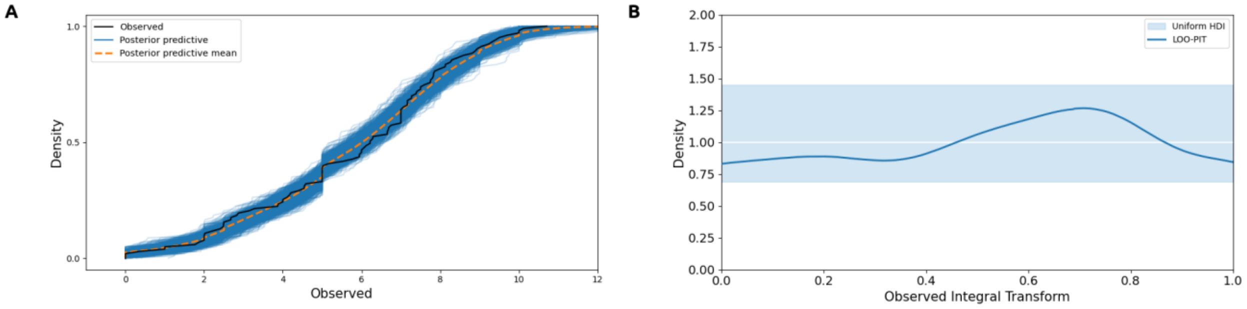

Figure S2: Posterior Predictive and Loo PIT. (A) Blue lines show 1000 posterior predictive samples' CD drawn from the model after fitting, the orange dotted line the mean of these CDs and the black line shows the CD for the data. **(B)** Blue line shows the log probability weighted sum of the probability integral transform (PIT) of observed data with respect to sampled data. The blue band shows the HDI of the PIT for samples. The PIT of the observed data is expected to be within the given HDI band if the model samples are similar to observed data.

We have seen that in this model using a StudentT likelihood instead of the Normal results in overall better performance and much well behaved pareto-k values which is shown in the first row of Fig.S3. The main problem seems to stem from the RLID dataset which demonstrates outlier behaviour. In the case of excluding this dataset, the behaviour of pareto values is generally better which is shown in the second row Fig.S3. In order to compare StudentT and Normal models, we carry out Pareto-smoothed importance sampling leave-one-out cross-validation. For any datapoint with non-acceptable pareto-k value (above 0.7), we complement this with an exact leave-one-out cross-validation. We again do this for the full dataset and with the dataset without RLID data. The results in Fig.S4 demonstrate that StudentT is useful when dealing with outlier data such as RLID but does not seem to be necessary when outliers are not present. In our case we prefer a more robust model who is able to deal with a reasonable amount of outliers and therefore go with the StudentT likelihood.

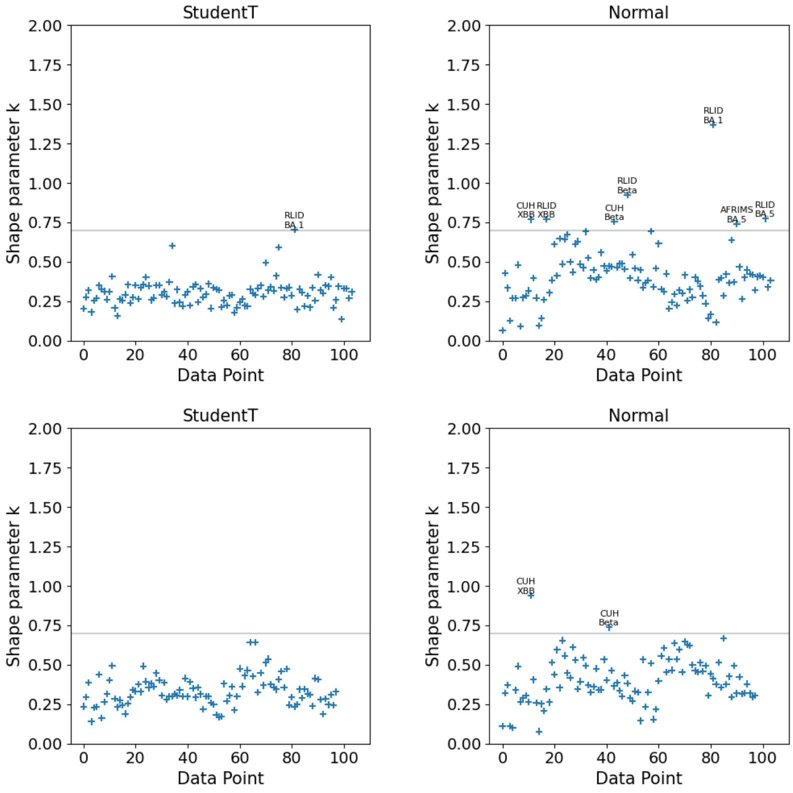

Figure S3: Pareto-k values for different models with and without RLID excluded. First row shows the pareto-k values for each observation obtained when a StudentT or Normal likelihood is used. Second row shows pareto-k values for the same models in the case RLID data is excluded. Data with pareto values below 0.5 are considered well behaving whereas for anything above 0.75, exact cross-validation must be performed during leave-one-out cross-validation (instead of pareto smoothed approximation). Observations with pareto k values higher than 0.7 line are labelled.

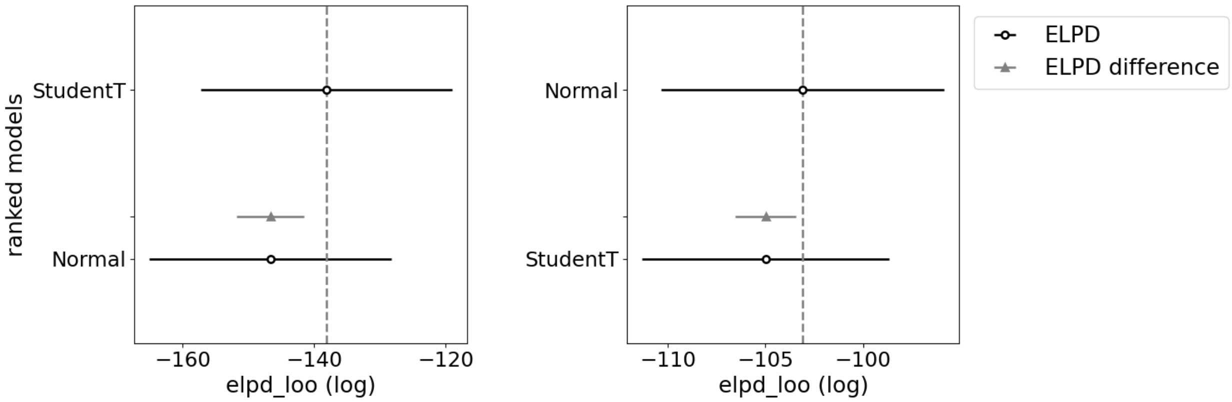

Figure S4: Comparison of StudentT and Normal models. Both figures show the comparison of StudentT vs Normal likelihood model, first one using the full dataset, the second one excluding the RLID data.

Additional Analysis for GMT and Offset Model.

By subtracting the dataset offsets from the raw data one can obtain raw data modulo the titer magnitude introduced due to other factors (such as assay type or titer computation method). This procedure is similar to computing fold-drops but instead of computing differences against a single antigen it is more akin to computing differences against the mean of titers. Similarly one can compute the average of dataset offsets across datasets and subtract it from the fitted gmt to get the gmt controlled for titer magnitude. Then one can compute pairwise differences of these quantities to see how compatible the results are which is shown in Fig.S5.

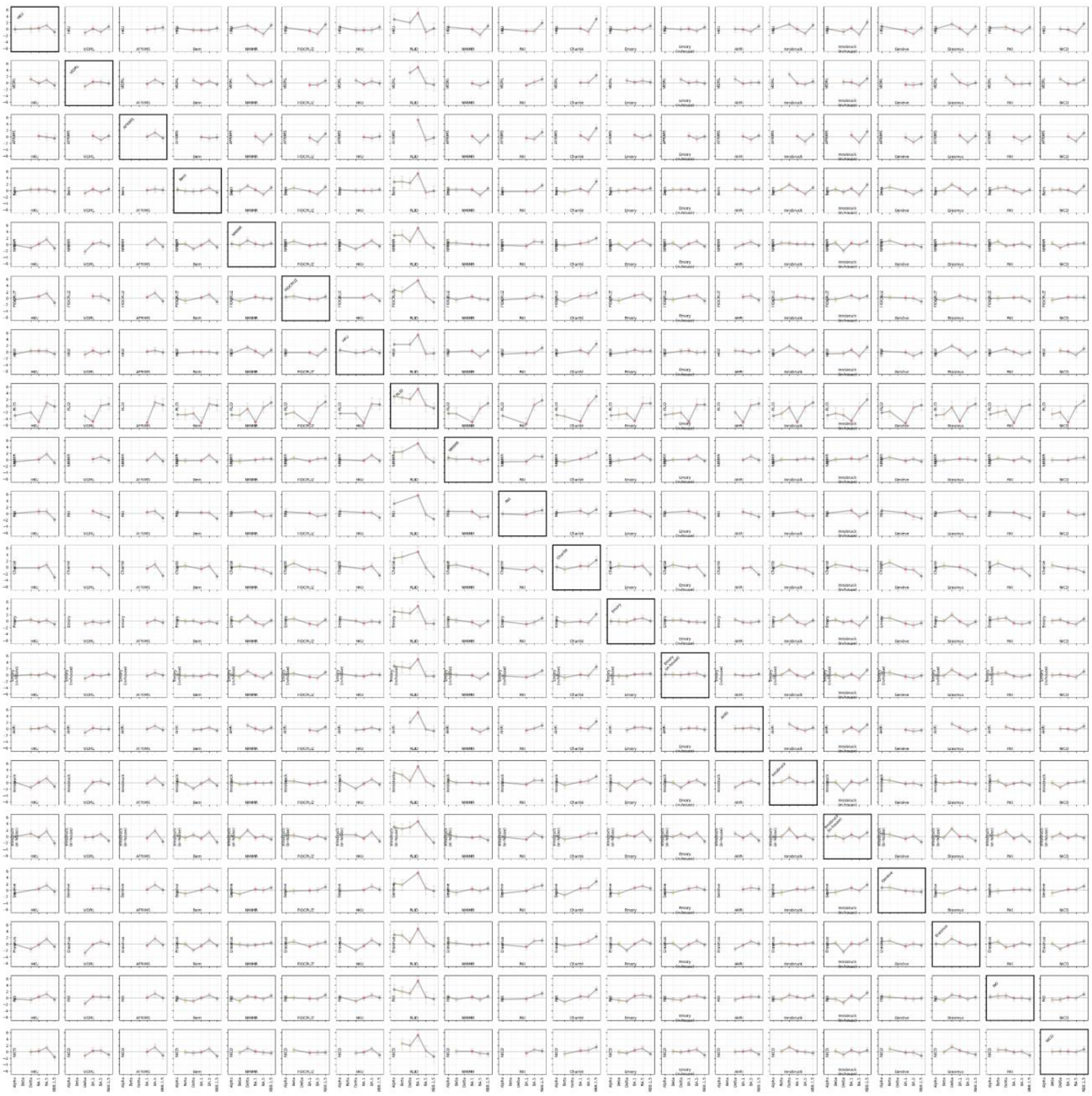

Figure S5: Comparison of fold-drops between each lab and fitted GMT. Diagonal axes show comparison of titers (controlled for dataset titer magnitude) for each dataset against the fitted GMT (controlled for overall titer magnitude) whereas off diagonal axes show the difference of fold-drops between the dataset indicated at the x-axis to the dataset indicated at the y-axis. Bars show 94% HDI intervals for pairwise differences.

Note that whereas one can use Fig.3 to get point estimates between titers of different variants, in order to get the correct 94% HDI one needs to compute them separately and this is what is shown in Fig.S6. The similar reasoning applies to pair-wise differences between assay-type offsets which is shown in Fig.S7

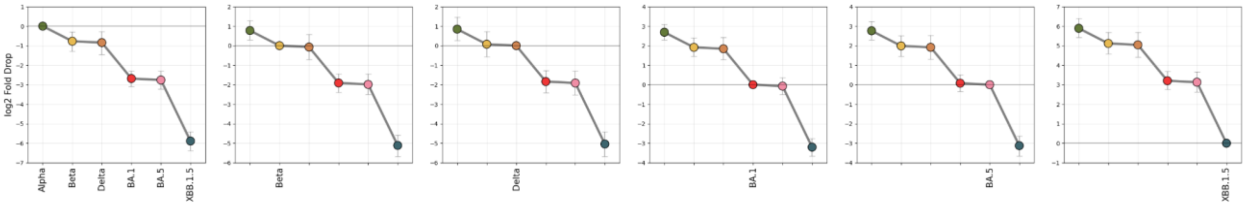

Figure S6: Fold-drops of titres with respect to each variant. Each plot shows the fold drop of titers with respect to one of the variants. The first plot is with respect to Alpha and the reference antigen for the others are shown in the x-axis. The bolder horizontal line shows the no fold drop line.

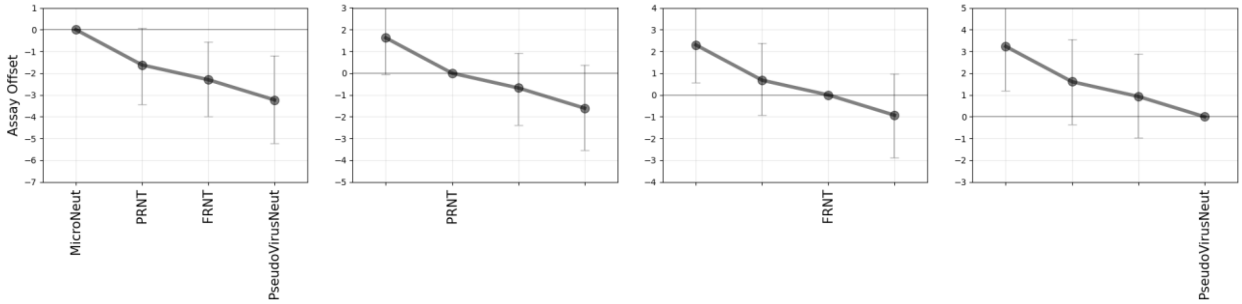

Figure S7: Pairwise difference between assay type offsets. Each plot shows the difference in assay type offsets with respect to one of the assays. The first plot is with respect to MicroNeut and the reference assay for the others are shown in the x-axis. The bolder horizontal line shows no difference.

Clustering Diagnostics

Determining the optimal number clusters. Model based selection of the number of clusters in the Bayesian context with methods such as loo is generally not possible [50]. Therefore, for determining the optimal number of clusters we look at the Silhouette scores, model logp values and the difference between fold-drops (with respect to first antigen) for cluster centres.

Fig.S8 shows how the mean sample log likelihoods, posterior probabilities and silhouette score changes as the number of clusters increase. In the classical setting, a silhouette score of higher than 0.5 is generally accepted as a passing score for the case of balanced clusters. In our limited experience and in the context of this problem the overall level of Bayesian silhouette scores seem higher than k-means silhouette scores and this could be due to several reasons (such as k-means silhouette score using distance, and not taking into account thresholded titres). From this comparison, it looks like the small gain in model efficiency after three clusters is not worth the large decrease in silhoutte score. We further analyse this in detail below.

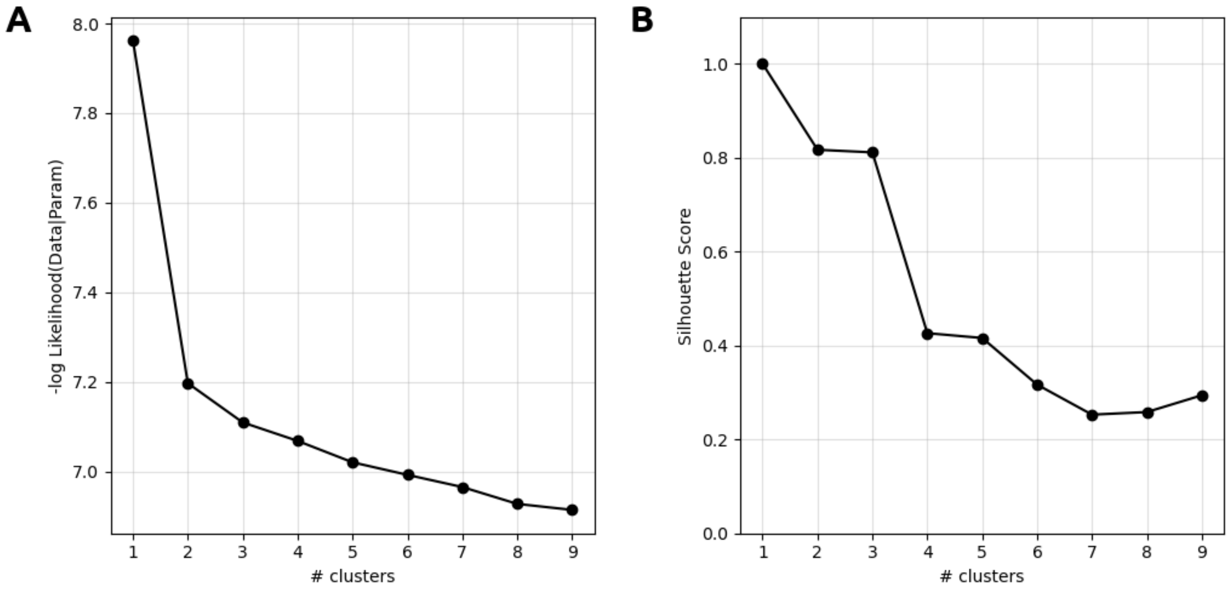

Figure S8: Likelihood and silhouette scores for different numbers of clusters. (A) The average log likelihood of the data given the sampled parameters is computed. **(B)** The average Bayesian Silhouette scores are computed as explained in the Clustering Titres section in SI.

To first determine that four clusters is the absolute maximum we want, we compare it to five clusters. Fig.S9 shows respectively the differences between cluster fold-drops when the number of clusters is four and five (the off-diagonal plots). Even when there are four clusters, it looks like the second cluster is not well separated from the first cluster (Fig.S9A, row 1, column 2). Addition of one more cluster creates an additional cluster centre which looks quite similar to the second cluster mentioned before (Fig.S9B row 2, column 3). In the case of three clusters, all of the clusters are well separated from each other (Fig.S10). The third cluster seems to also generate a biologically relevant group when one looks at the categories of encounters belonging to this group vs others (Fig.4, Fig.S13). On the other hand looking at Fig.S8, one sees that the major improvement for model likelihood and posterior probability comes from addition of the second cluster. The third cluster, whereas only seems to provide a modest improvement, does not lead to deterioration in Silhouette Score and does capture a biologically relevant group which comes from BA.1 convalescent sera. On the other hand, addition of a fourth cluster only seems to divide the first cluster into two parts since its addition decreases silhouette score quite substantially and none of the sera in the split first two clusters can be confidently assigned (with an error threshold of 0.1). Therefore we decide to go with a total of three clusters. If instead two clusters are chosen, then the members of the third cluster are categorised as outliers which could have been preferred if there were no metadata available for these sera and the biological relevance of this cluster could not be verified.

Note that the third cluster in Fig.S9A, Fig.S10 and the fourth cluster in Fig.S9B have relatively large uncertainty bars because these are clusters where data against Alpha antigen are almost completely missing so fold drops are relatively uncertain (mostly determined by priors). These clusters are nevertheless well separated from other clusters in terms of their BA.1 titres and large bars are remedied if fold-drops are measured for instance against Beta (not shown).

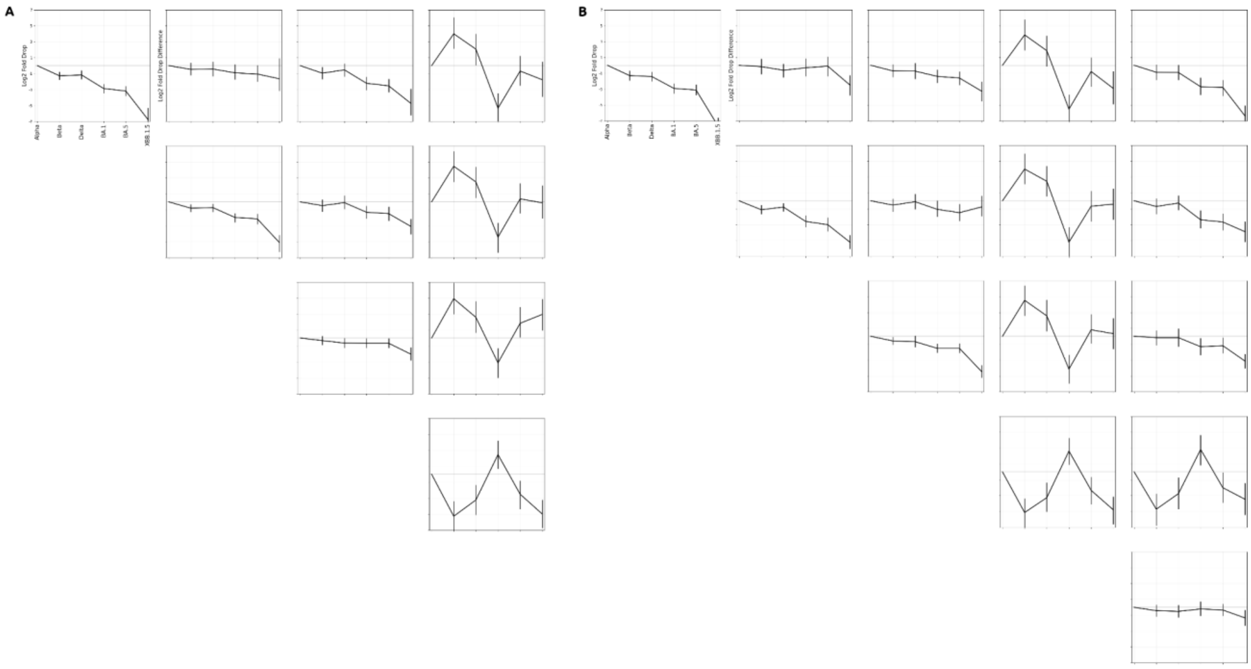

Figure S9: Differences between cluster centre fold-drops for four and five clusters. (A) Shows a comparison of fold drops for different cluster centres when the number of clusters is four whereas **(B)** shows it when it is five. The diagonal plots show the centred log titres for identified clusters and off-diagonal terms show the sampled difference between cluster centres. The bars represent the 94% HDI.

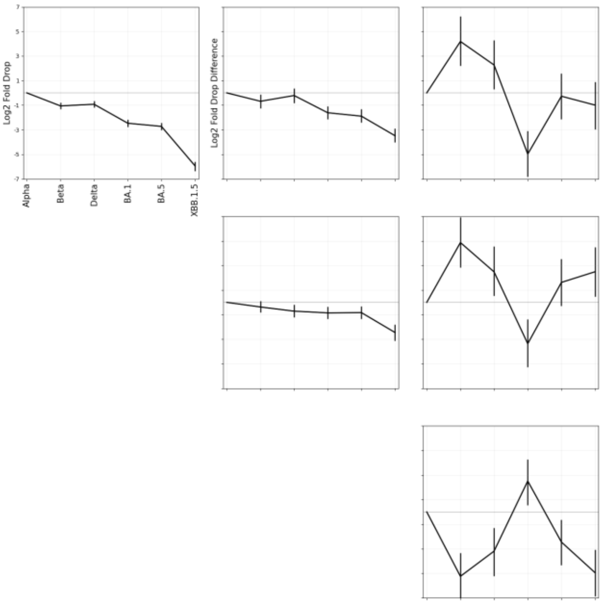

Figure S10: Differences Between Cluster Centre Fold-drops for Three Clusters: This figure shows the cluster centre comparisons as in Fig.S9 except that it is for three clusters. Diagonal plots show the lo2 fold drop for each centre whereas the off-diagonal plot show shows the pairwise comparison between different centres.

Convergence Diagnostics. In this section we provide the necessary plots required to verify that there were no convergence issues and the fitted cluster centre posterior does not suffer from multi-modality and mixing.

The unimodal posteriors for cluster centres and for the rank and the "hairy caterpillar" look of the trace plots aligned along a stable mean (rather than step-wise jumps between different means) is the indication of good chain convergence (Fig.S11).

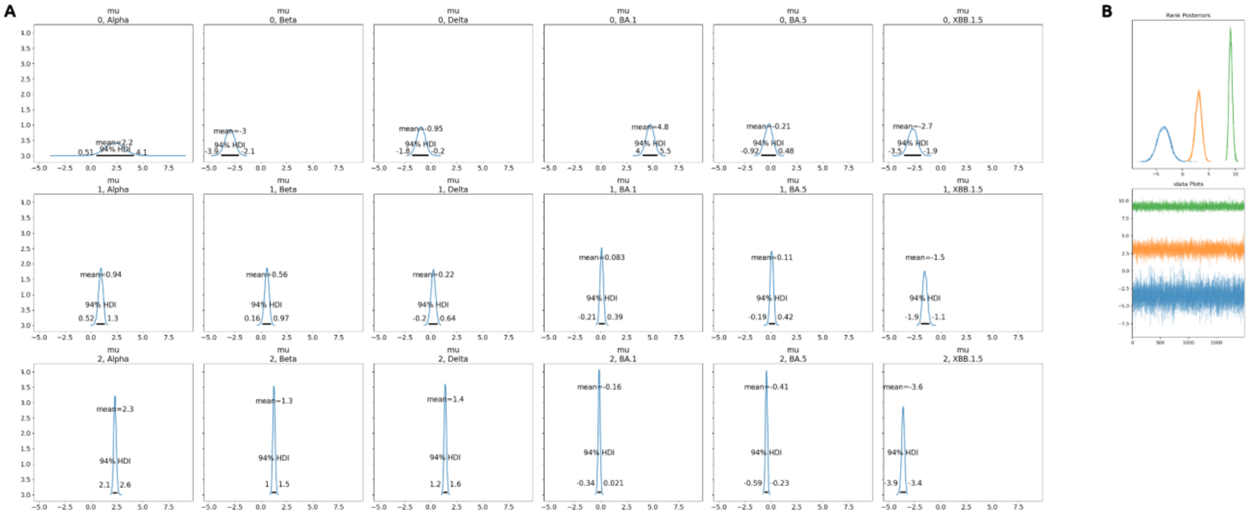

Figure S11: Posterior Distributions for Cluster Centres and Rank, Trace Plot for Rank. (A) This shows the posterior distribution for the cluster centres (non-outlier) when the number of clusters is three. **(B)** This plot shows the posterior distribution and the trace plot for the rank parameter (see Mathematical Methods – Clustering Titers in SI).

Euclidean k-means Clustering vs Bayesian Clustering. Fig.S12 shows the three clusters obtained from the k-means algorithm with Euclidean distance metric. The second cluster is an example of erroneous cluster assignments because k-means with Euclidean distance metric can not deal with thresholded titers. Indeed this cluster contains sera with 3+ WT vaccinations and thresholded titres against BA.1, BA.5 and XBB.1.5 (emphasised green lines with straight ends for the last two-three variants). This makes them seem like more cross-reactive than they really are and therefore are grouped together with a bunch of sera that show relatively high reactivity to BA.1, BA.5, XBB.1.5. In reality these sera are probably more like the first cluster. Indeed in comparison, Bayesian clustering (Fig.4A or Fig.S14) groups them with a cluster that looks like the first one (shown by the subset of the green lines with straight ends in the first group). The identified cluster centres for the first and second group in k-means also under-estimates the XBB.1.5 fold-drops from Alpha (as also can be seen in Fig.S15). Another alternative approach could be to introduce to k-means a metric that takes into account thresholding (possibly the exponential of the log likelihood defined by a censored normal) and using standard deviations of each cluster to define probability of a data belonging to a cluster. We have not investigated this line of approach.

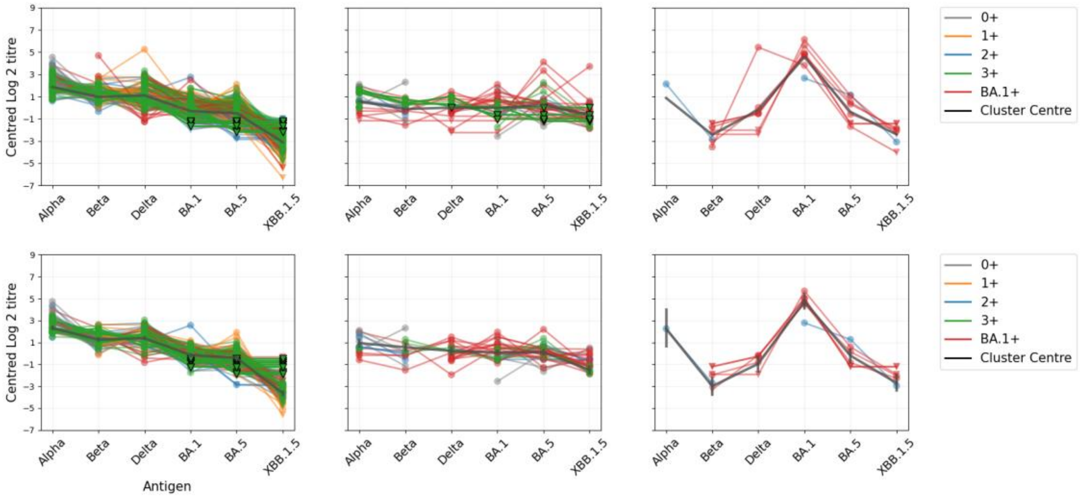

Figure S12: Clusters Obtained with Euclidean k-means. The first row shows the three clusters obtained from applying k-means clustering the labs' collated serum data whereas the second shows the result obtained from the Bayesian clustering model. The figure manual is identical to that of Fig.4A except that 3+ sera with flat ends are emphasised to facilitate comparison between two models. The darker line represents the cluster centre.

Additional Analysis. In the figure below, we show a more refined variant breakdown of the clusters shown in Fig.4.

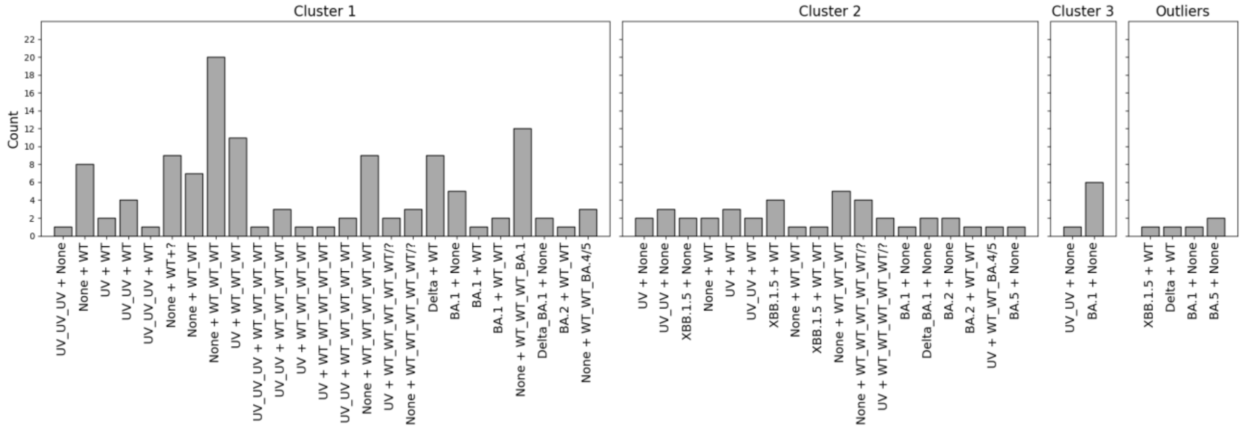

Figure S13: Serum Encounter Categories of Clusters. This figure shows the variant breakdown of the clusters identified in Fig.4. It shows the encounter as infections + vaccinations. UV stands for Unidentified Variant and None stands for no recorded infection or vaccination. Note that in many cases these meta data are collected simply based on personal declaration and not a strict follow-up. Also note that this shows the breakdown of serum whose probability of belonging to any other cluster is less than 0.1.

After the data is clustered, we separate each group into two subsets. One of the subsets is made up of elements for which the probability of belonging to any other cluster is higher than 0.1 whereas the other group is made of data for which it is lower than 0.1. This group is shown in Fig.S14.

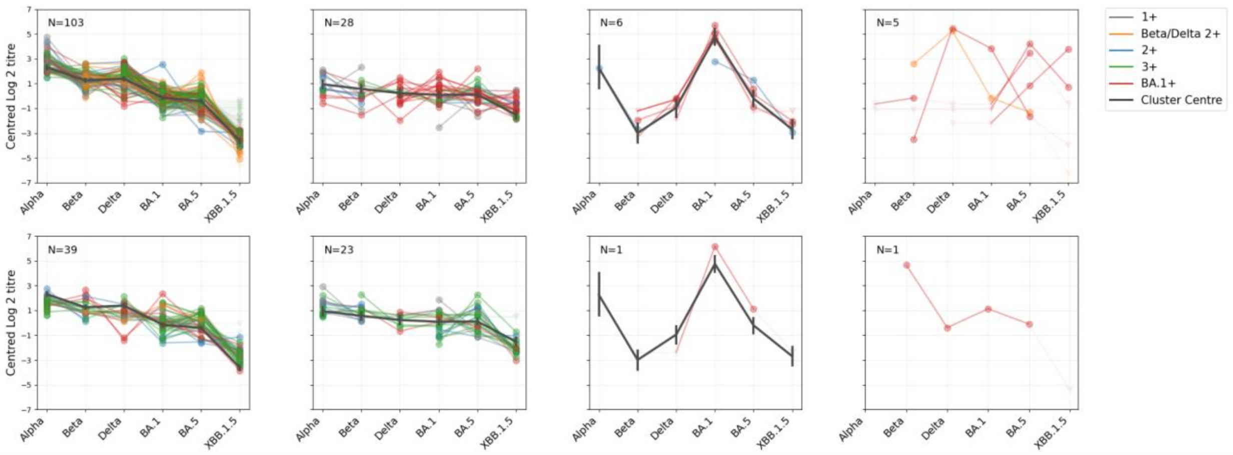

Figure S14: Data in Each Cluster Grouped According to Confidence in Identification. The first row of this plot is identical to Fig.4A and shows the data for which the probability of belonging to any other cluster is less than 0.1. The second row shows the data for which the probability of belonging to any other cluster is higher than 0.1.

Comparing the fold-drops obtained from the cluster centre for the first cluster to non-parametric point estimates obtained from data reveals an under-estimation of fold drop for XBB.1.5 when point estimates are used (compare black vs green in Fig.S15). Since there are many sera in the first group which do not have titres measured against Alpha (about %50) their point estimate fold drops against Alpha can not be computed. To have a more fair comparison, we have used fold drop against Beta for comparison. One sees that setting thresholded titers to threshold -1 (red line) remedies this to some extent (the normal rule of thumb is to reduce them by -1 for HI titers which represent a discrete spectrum of rounded down titers and to leave it as the threshold value for neutralisation type titers where the spectrum of titers is continuous). The -1 value would likely depend on percent of thresholded titers vs non thresholded titers for a given variant. In this case about %65 percent of the titers for XBB.1.5 were thresholded.

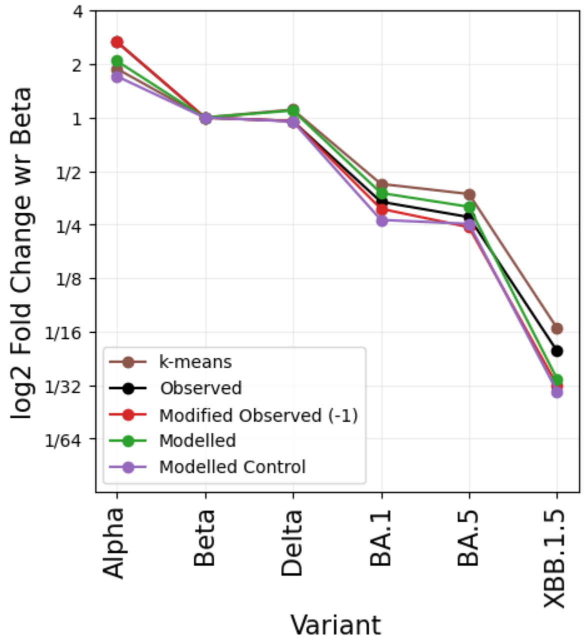

Figure S15: Comparison of fold drops obtained via various methods. Black line shows fold drops (against Delta) obtained from the data corresponding to members of the first cluster shown in Fig.S14 first row, by first computing fold drops for every data and then averaging them. Due to many sera with missing titres against Alpha, Beta was chosen to

be the reference strain in this plot. The red line shows the average of the data in the same way but lower thresholded titers are reduced by -1 instead of using the threshold as the value. The green line shows the fold drop obtained from the cluster centre for this group obtained in clustering analysis of the data and finally the purple line shows the fold drops obtained from modelling the NIBSC IS 21/338.

Landscape Diagnostics & Additional Analysis

Convergence and Goodness of Fit Diagnostics. The posterior predictive plots in Fig.S16 indicate reasonably high good fit (noting that for group three there is relatively little data so the posterior predictive plots are more spread and data cumulative distributions (CDs) are more wobbly). The loo pit plot indicates some possible problems in the case of the first group. This group has enough data to split the ppc and loo pit into separate antigens which is shown in Fig.S17. This plot suggests that the main problem occurs from XBB.1.5 and possibly Delta. The shape of the loo-pit curve for XBB.1.5 suggests that there is a bias for fitted titres of XBB.1.5 to be smaller than observed which could suggest that a more human serum compatible position for XBB.1.5 is actually slightly further in. For Delta the situation is opposite and a more human serum compatible position might be slightly outwards. Note that the map used in fitting the landscapes is a hamster serum map made for a separate study. Moreover since XBB.2 was the only XBB variant present in the map, its position was used for XBB.1.5. It is possible that there might be slight inconsistencies between the human serum used in this study and the positioning of the antigens in this map. Investigating this further is beyond the scope of this manuscript.

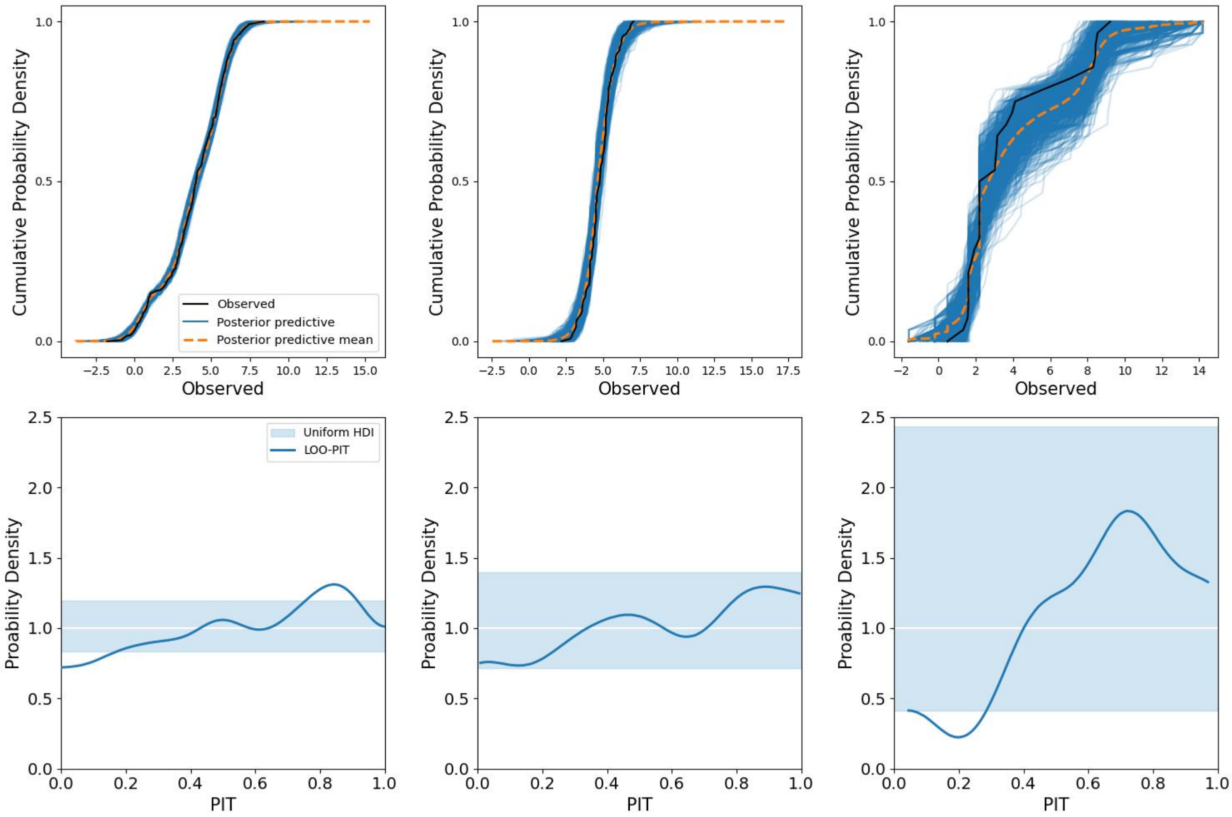

Figure S16: Posterior Predictive and LOO PIT plots for the Landscapes. Blue lines in the first row show 1000 posterior predictive samples' CD drawn from the model after fitting, the orange dotted line the mean of these CDs and the black line shows the CD for the data. Columns correspond to the landscapes fitted to the groups identified by clustering. Blue line in the second row shows the log probability weighted sum of the probability integral transform (PIT) of observed data with respect to sampled data for each of the groups. The blue band shows the HDI of the PIT for samples. The PIT of the observed data is expected to be within the given HDI band if the model samples are similar to observed data.

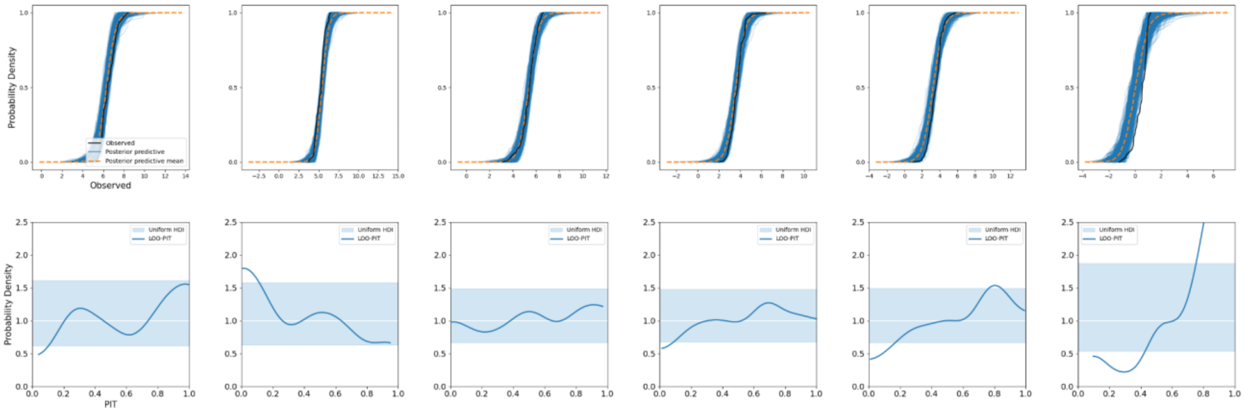

Figure S17: Posterior Predictive and LOO PIT plots for the Landscapes for the first group split into antigens. The first row shows the first posterior predictive plot in Fig.S16 split into antigens Alpha, Beta, Delta, BA.1, BA.5, XBB.1.5. The second row shows the first loo pit plot in Fig.S16 split into antigens in the same order.

Fig.S18 shows the posterior density plots for the combined landscape titer values. This is the value given by $F(h_s - d(\mu_s, c_i) \times \beta_s + \xi_j)$ which reflects the landscape titer per each antigen. The posterior plots and traces indicate good convergence.

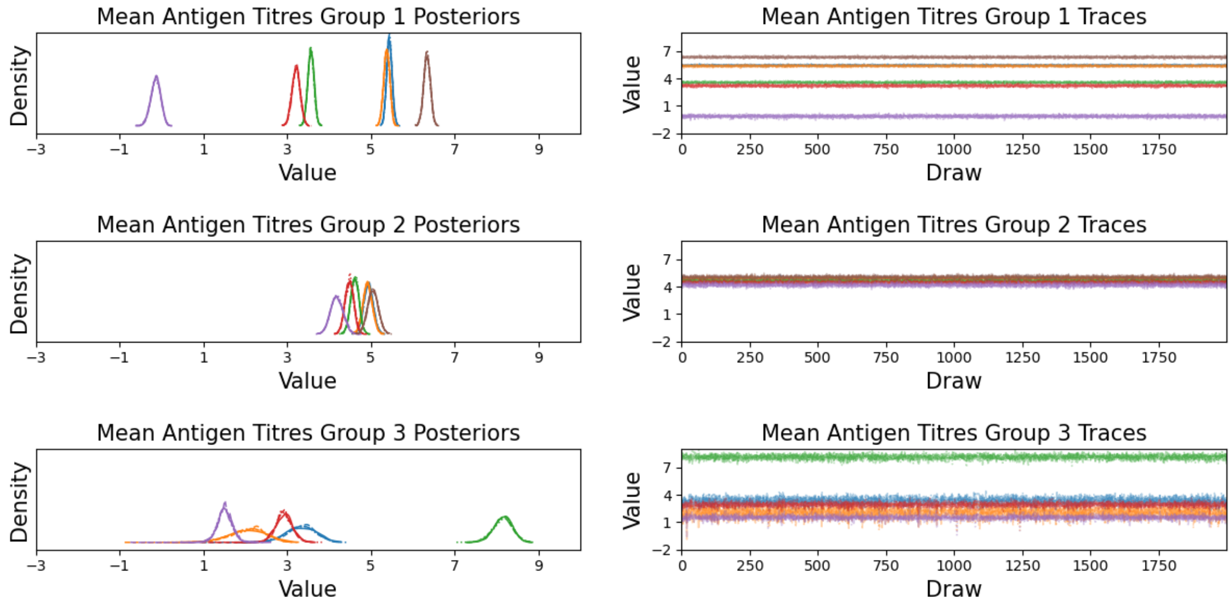

Figure S18: Posterior and Trace Plots For Mean Titres of Antigens. Each row above shows the posterior density of mean titers of antigens and its trace plot. Each curve represents a separate antigen. The third row does not have antigen Alpha because it is a cluster where titres against that antigen were not measured in the data.

Determining the Optimal Number of Cones. In order to determine the number of clusters, we compare each cluster modelled with one and two clusters using LOO (leave one-out cross validation) estimate function in [51]. From Fig.S19, we see that two cones is better for the first landscape whereas one is for the other too. Note that we have relatively few antigens in the map (most of which are relatively collinear) and a more precise determination of number cones could benefit from a larger number of antigens. Although not shown here, almost all of the pareto-k values for groups 1 and 2 are within range -inf,0.5 (good) with only a couple in 0.5-0.7 (OK) and none in 0.7,inf (bad) range. There is only a single bad pareto value in group 3 with two cones but given that the elpd difference standard deviation was small enough give a decisive result and there are relatively few observations in this group and finally that from the metadata we can confidently guess that this group is a single cone group (since convalescent with a single variant), we have not pursed exact leave out cross-validation for the data with bad pareto-k values.

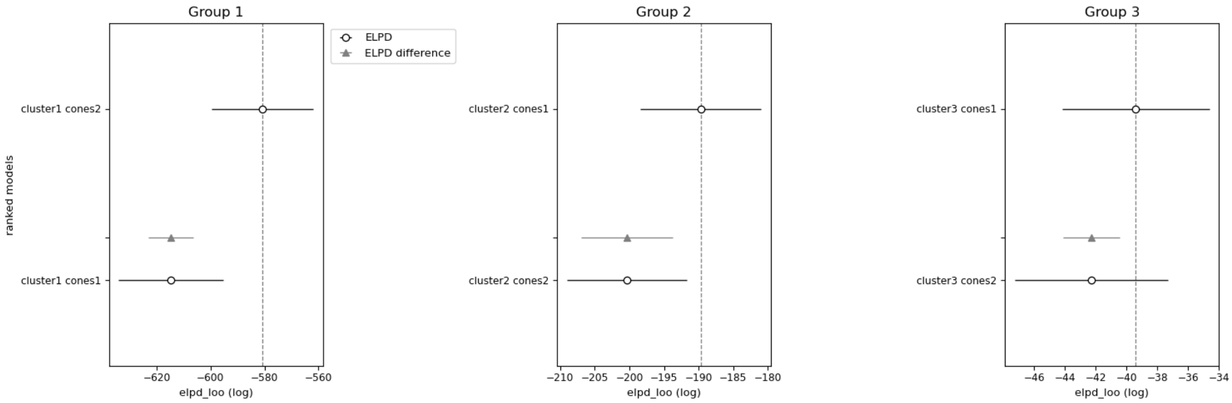

Figure S19: Model Comparison for Different Number of Cones. Each plot represents landscapes fitted to a cluster with one or two cones. Models with larger ELPD are better. Bars show significance level and an ELPD difference bar not intersecting the dashed line indicates that the first model is significantly better.

Note that in fitting landscapes to the data, the model requires a mixture of apex coordinates and heights. For all the cases, the mixture of coordinates and heights were weighted combinations of respectively Alpha, BA.1, BA.5 map coordinates and mean data heights with a standard deviation of 0.5 for both coordinates and heights. Guided by the plots Fig.4A when fitting a single cone, the following weights were used for the three clusters: [1, 0, 0], [1, 0, 0], [0, 1, 0]. For instance [1, 0, 0] means that the apex coordinate will be around Alpha and its height will be similar to the average titer of the group for Alpha. When fitting two cones the following weights were used: {[1,0,0], [0, 1, 1]}{Karim, 2024 #2;Khan, 2023 #1}, {[1,0,0], [0, 1, 1]}, {[0, 1, 0], [1, 0, 1]}. For instance {[1,0,0], [0, 1, 1]} means the first cone of this cluster will be around Alpha and the second cone will be around BA.2,BA.5.

Additional Analysis. For sampling the 94% HDI landscape fitted to data, we define a grid of side-length 0.1 (compared to the unit of the map) and sample the posterior distribution of $F(h_s - d(x, c_i)$

x $\beta_s + \xi_j$) on this grid where x represents the grid points. The 94% HDI of these samples show us the significant amount of coverage one can get from the landscape as shown in Fig.S20.

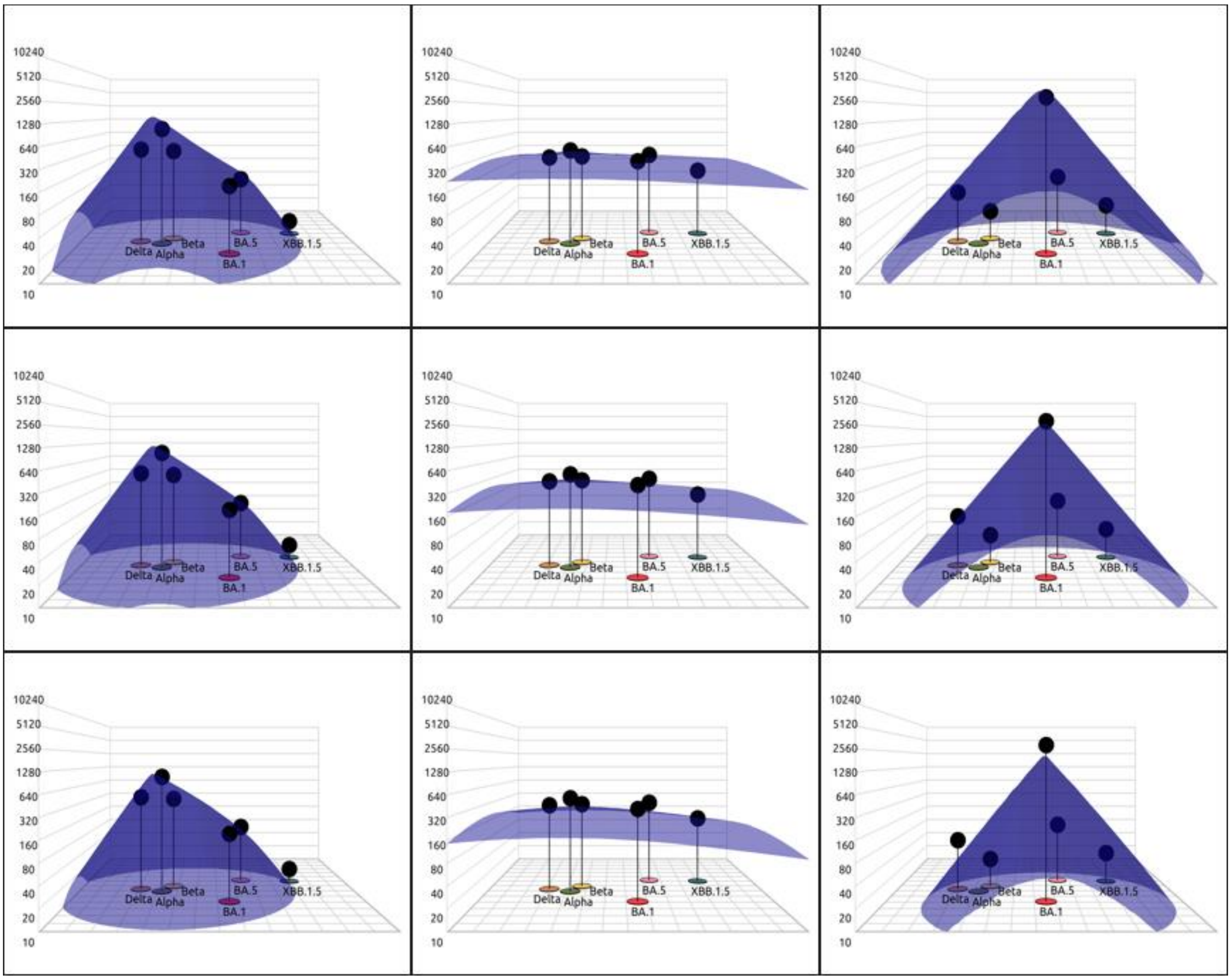

Figure S20: Landscapes Fitted to Three Groups and Their HDI. Each column shows a landscape fitted to the group of clusters shown in Fig.4A. First row shows the higher end of the 94% HDI, the second row the mean and the third row the lower end of the 94% HDI.

The map used for making the landscapes is shown in Fig.S21 [19]. Since this map does not contain XBB.1.5, the position of XBB.2 was used instead when making landscapes.

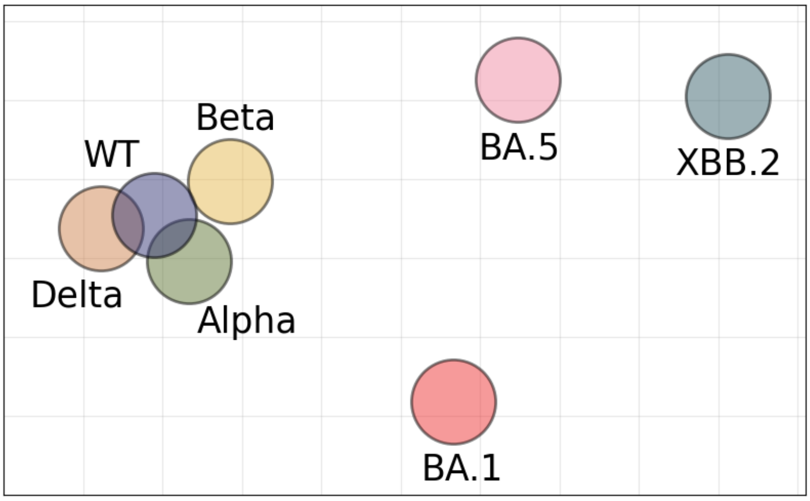

Figure S21: The Antigenic Map Used for Landscapes. The map shows all the variants involved in the study (modulo XBB.2 instead of XBB.1.5) and wild-type (D614G) as reference. The original map, which contains more variants and sera, can be found in [19].

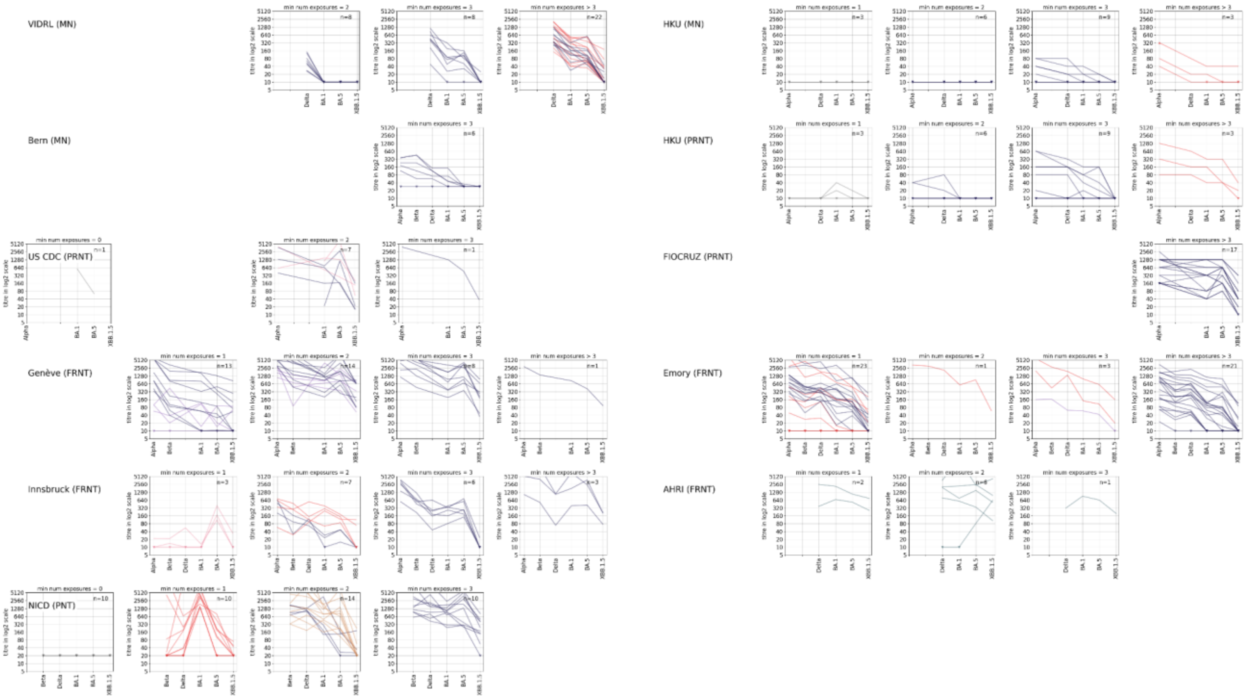

Figure S22: The raw titres for human sera obtained from labs. Each column represents the minimum number of encounters of the sera in the given column. First column is zero encounters and the fourth column is four or more, where an encounter denotes infection or vaccination. Colouring of the lines indicate encounter or vaccination variant (when available) and the colour legend is as follows: No encounters: Grey, Unidentified Variant: Purple, other antigens: same as the colour given in map Fig.S21. If a serum has multiple encounters, the variant which is chronologically last is taken into account for colouring.

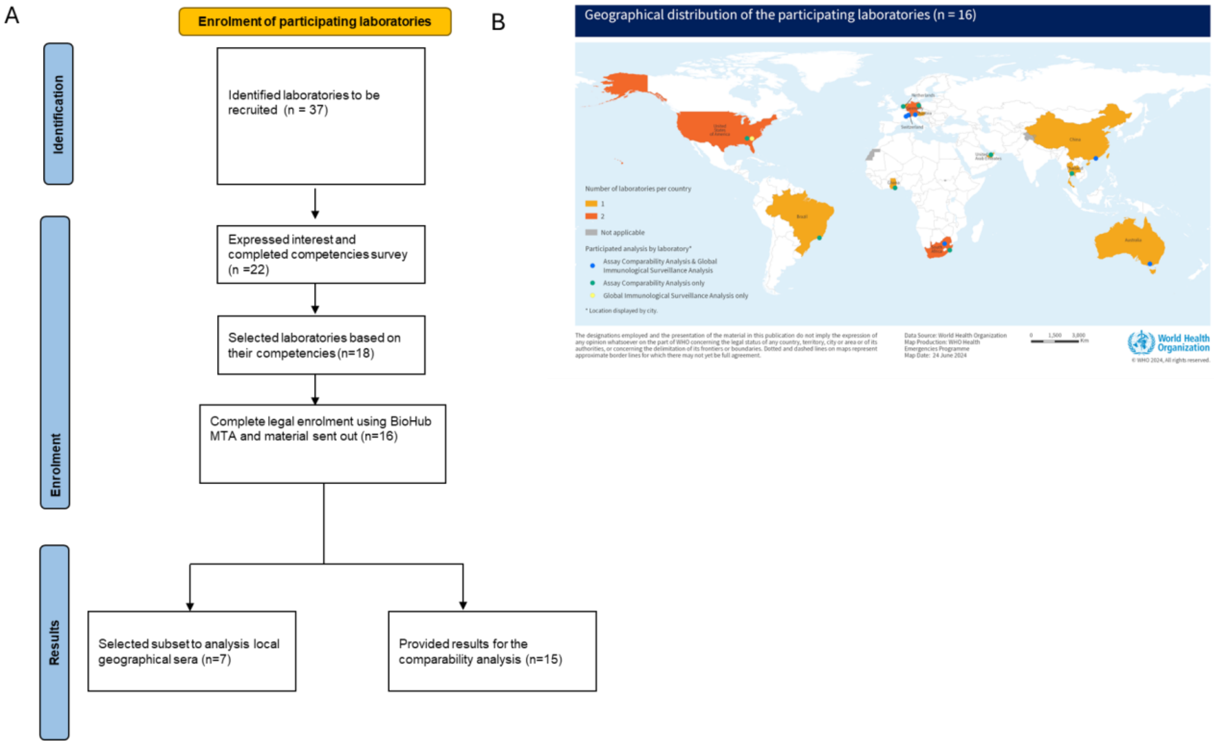

Figure S23: Laboratory enrollment process and geographical representation. A. laboratory enrollment process. B. Geographical distribution of the participating laboratories.

Comparing the WHO/Emory/Innsbruck stocks used in some of the titrations (Fig.S24), we have seen differences in the following positions: 15 (F/F/L), 879 (A/A/S) for Beta, 153 (G/E/G), 282 (X/S/N), 771 (A/A/S), 1167 (V/G/G), 1219 (V/G/G) for Delta, 73 (T/T/I) for BA.5 and 573 (T/T/I) for XBB.1.5.

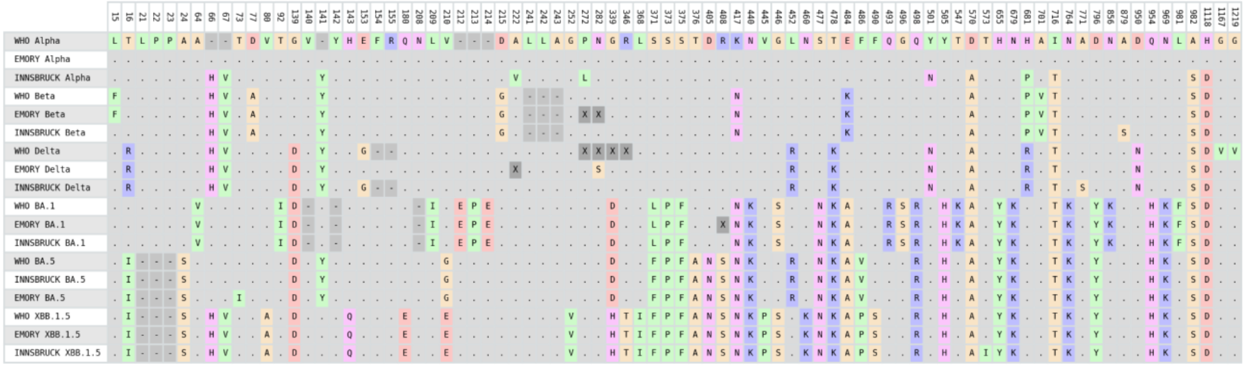

Figure S24: Comparison of spike sequences for the variants used in this study . This table displays the spike positions for which variability was observed among the variants used in this study. The colouring is according to the Lesk scheme [52] which categorises amino acids based on physico-chemical similarity as follows; yellow: small nonpolar, green: hydrophobic, magenta: polar, red: negatively charged, blue: positively charged. Deletions are shown by - whereas unknown amino acids are shown by X. A dot indicates that the amino acid is identical to the amino acid of WHO Alpha which is shown in the first row.

CODE	NAME	SHORT NAME	ASSAY-TYPE	GROWTH-CELL	ASSAY-CELL	REPS	VIRUS-TYPE	LLD	ULD	TITER-METHOD	NOTES
**AFRIMS_TH_SEARO**	US Army Medical	AFRIMS	MN	Vero E6	Vero E6	8	live	10	163840	titer computed using log	NA

	Directorate - Armed Forces Research Institute of Medical Sciences									probit a?lysis	
**EMS_NL_EURO**	Erasmus Medical Center	Erasmus	FRNT	Calu-3	Vero-TMPRSS2	4	live	20	None	GraphPad nonlinear regression to calculate 50% reduction in infected cells	NA
**VIDRL_AU_WPRO**	Victorian Infectious Diseases Reference Laboratory	VIDRL	MN	Vero E6-TMPRSS2 / Calu-3	Vero E6-TMPRSS2	4	live	10	2560	NA	NA
**RLID_AUE_EMRO**	Reference Laboratory for Infectious Disease - Abu Dhabi	RLID	PRNT	Vero E6	Vero E6	2	live	10	320	BioTek Gen5 was used for curve fitting and titer computation	NA
**FIOCRUZ_BS_PAHO**	Fundação Oswaldo Cruz - Rio de Janeiro	FIOCRUZ	PRNT	Vero E6	Vero CCL81	2	live	10	5120	titer was the highest serum dilution that reduced in 90% the number of viral plaques in relation to non-serum controls	NA
**USCDC_PAHO**	Center for Disease Control - US	US CDC	PRNT	NA	VeroE6/TMPRSS2/ACE2	NA	live	10	7290	graph-pad was used for calculating titers.	WA-1 instead of Alpha and ND10 titers because of stock problems
**NMIMR_GH_AFRO**	Noguchi Memorial Institute for Medical Research	NMIMR	MN	Vero E6 TMPRSS2	Vero TMPRSS2	2	live	40	5120	GraphPad was used to estimate the ND50 titres	NA
**NMIMR_GH_AFRO**	Noguchi Memorial Institute for Medical Research	NMIMR	PRNT	Vero E6 TMPRSS2	Vero TMPRSS2	2	live	40	5120	GraphPad was used to estimate the ND50 titres	NA
**AHRI_ZA_AFRO**	African Health Research Institute	AHRI	FRNT	Vero E6 TMPRSS2	Vero E6 - TMPRSS2	1 (own sera) / 4 (control)	live	25	3200	FRNT determined using MATLAB	NA
**CUH_DE_EURO**	Charité – Universitätsmedizin Berlin	Charité	PRNT	Vero E6	Vero E6	2	live	40	10240	titers computed via neutcurve package	NA
**UHG_CH_EURO**	Hôpitaux Universitaires de Genève	Genève	FRNT	Vero-E6/TMPRSS 2	Vero E6/TMPRSS	2	live	10	81920	titers computed via 4 parameter curve fitting	NA
**EVC_US_PAHO**	Emory Vaccine Center - Emory University	Emory	FRNT	Vero E6/TMPRSS 2	VeroE6 TMPRSS2	2	live	10	43740	titer computed using 4 parameter curve fitting	NA
**FIVI_CH_EURO**	Institute of Virology and Immunology - Universität Bern	Bern	MN	Vero E6/TMPRSS 2	Vero E6/TMPRSS2	3	live	28.3/56.6	2560	titers computed using Spearman and Kärber method	NA
**MUI_AT_EURO**	Medizinische Universität Innsbruck	Innsbruck	FRNT	Vero-TMPRSS2/ACE2	Vero-TMPRSS2/ACE2	2	live	16	163840	titers computed using graph-pad non-linear regr.	NA
**NICD_SA_AFRO**	National Institute for Communicable Diseases of South Africa	NICD	PNT	Lentivirus produced in 293T/17	293T/ACE2	2	pseudo (lentiviral bb)	20	81920	GraphPad was used to estimate the ND50 titres	NA
**RKI_DE_EURO**	Robert Koch-Institut	RKI	PRNT	no virus growth done	Vero E6	3	live	10	20480	titer was lowest serum dilution that yields less than %50 of initial	Beta and Delta did not produce any plaques
**RKI_DE_EURO**	Robert Koch-Institut	RKI	PNT	Lentivirus	HT1080-ACE2	3	pseudo	40	10800	titers computed using	Delta Pseudo lacks G142,

				produced in HEK293T	cells					graph-pad non-linear regr.	G1167 and G1219
**SPH_HK_WPRO**	School of Public Health - University of Hong Kong	HKU	MN	Vero E6 TMPRSS2	Vero E6 TMPRSS2	4	live	10	320	titer was the highest serum dilution that completely protected the cells from CPE in half of the wells	NA
**SPH_HK_WPRO**	School of Public Health - University of Hong Kong	HKU	PRNT	Vero E6 TMPRSS2	Vero E6 TMPRSS2	2	live	10	10240	titer was lowest serum dilution that yields less than %50 of initial	NA

Table S1: The assay information for the labs involved in the study.

**Variant**	**Accession Number**
WHO Alpha	EPI_ISL_3147384
Innsbruck Alpha	EPI_ISL_3305837
Emory Alpha	EPI_ISL_751801
WHO Beta	EPI_ISL_2401142
Innsbruck Beta	EPI_ISL_17528983
Emory Beta	EPI_ISL_890360
WHO Delta	EPI_ISL_5394579
Innsbruck Delta	EPI_ISL_2290769
Emory Delta	EPI_ISL_2457061
WHO BA.1	EPI_ISL_7456457
Innsbruck BA.1	EPI_ISL_17528984
Emory BA.1	EPI_ISL_7171744
WHO BA.5	EPI_ISL_12268493.2
Innsbruck BA.5	EPI_ISL_13666092
Emory BA.5	EPI_ISL_13512579
WHO XBB.1.5	EPI_ISL_16760602
Innsbruck XBB.1.5	EPI_ISL_17077093
Emory XBB.1.5	EPI_ISL_16026423

Table S2: The accession numbers for sequences of variants used in this study.

**Infection + Vaccination**	**Count**
None + WT_WT_WT	39
None + WT_WT	27
BA.1 + None	19

**Infection + Vaccination**	**Count**
UV + None	4
unknown + WT_WT	4
BA.5 + None	3

None + WT_WT_WT_BA.1	18
UV + WT_WT_WT	12
None + WT_WT_WT_WT_WT/?	12
None + WT+?	12
None + None	11
None + WT	11
Delta + WT	10
None + WT_WT_WT_WT_WT	10
UV + WT	9
UV_UV + WT	8
BA.2 + WT_WT	6
BA.2 + None	6
XBB.1.5 + WT	6
UV_UV + None	5
UV + WT_WT_WT_WT_WT/?	5
Delta_BA.1 + None	4
UV + WT_WT_BA.4/5	3
UV_UV + WT_WT_WT_WT	3
None + WT_WT_BA.4/5	3
unknown + WT_BA.4+BA.5	3
UV + WT_WT_WT_WT	2
UV_UV + WT_WT_WT_WT_WT	2
BA.1 + WT_WT	2
XBB.1.5 + None	2
UV_UV_UV + WT	1
UV_UV_UV + WT_WT_WT_WT	1
UV + WT_WT_WT_WT_WT	1
XBB.1.5 + WT_WT	1
UV_UV_UV + None	1
BA.1 + WT	1
unknown + WT_WT_WT	1

Table S3: The infection and vaccination categories of sera used in the study. This table shows the infection and vaccination categories of the in-house sera provided by the labs. The format is Infection + Vaccination. The information was extracted from the meta data made available from each of the labs and both the raw and processed forms of the metadata can be found in the code repo [14]. UV stands for unidentified variant and WT/? stands for unspecified bivalent.
